# Negative feedback buffers effects of regulatory variants

**DOI:** 10.15252/msb.20145844

**Published:** 2015-01-29

**Authors:** Daniel M Bader, Stefan Wilkening, Gen Lin, Manu M Tekkedil, Kim Dietrich, Lars M Steinmetz, Julien Gagneur

**Affiliations:** 1 Computational Genomics, Gene Center, Ludwig Maximilians UniversityMunich, Germany; 2 European Molecular Biology Laboratory, Genome Biology UnitHeidelberg, Germany; 3 Stanford Genome Technology CenterPalo Alto, CA, USA; 4 Department of Genetics, Stanford University School of MedicineStanford, CA, USA

**Keywords:** buffering, canalization, cis regulation, feedback, trans regulation

## Abstract

Mechanisms conferring robustness against regulatory variants have been controversial. Previous studies suggested widespread buffering of RNA misexpression on protein levels during translation. We do not find evidence that translational buffering is common. Instead, we find extensive buffering at the level of RNA expression, exerted through negative feedback regulation acting in trans, which reduces the effect of regulatory variants on gene expression. Our approach is based on a novel experimental design in which allelic differential expression in a yeast hybrid strain is compared to allelic differential expression in a pool of its spores. Allelic differential expression in the hybrid is due to cis-regulatory differences only. Instead, in the pool of spores allelic differential expression is not only due to cis-regulatory differences but also due to local trans effects that include negative feedback. We found that buffering through such local trans regulation is widespread, typically compensating for about 15% of cis-regulatory effects on individual genes. Negative feedback is stronger not only for essential genes, indicating its functional relevance, but also for genes with low to middle levels of expression, for which tight regulation matters most. We suggest that negative feedback is one mechanism of Waddington's canalization, facilitating the accumulation of genetic variants that might give selective advantage in different environments.

## Introduction

Regulatory genetic variants play a major role in phenotypic variation and evolution. Most genetic variants are non-coding and they are the major driver of speciation (King & Wilson, 1975). Moreover, non-coding genetic variants represent the majority of genetic associations with common diseases (Gibson, 2009; Manolio, 2010). Hence, given the potential phenotypic impact of regulatory variants, biological mechanisms conferring robustness against their effects are expected.

**Figure 1:**
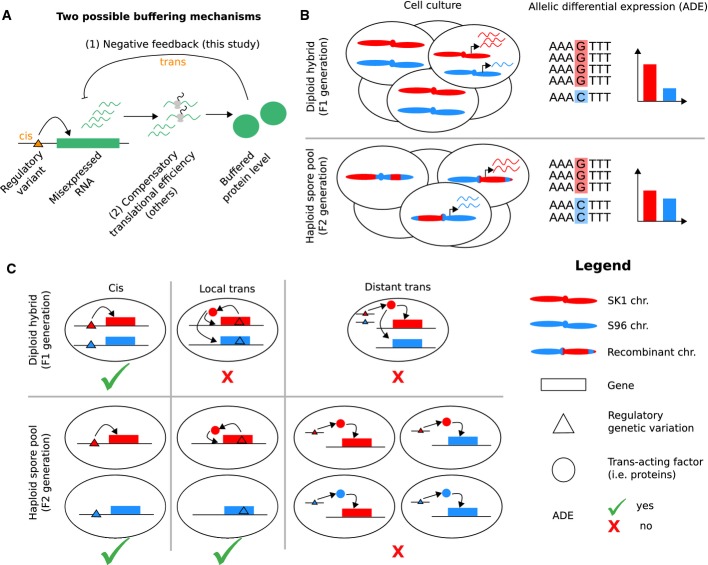
Tested hypothesis and experimental design  Effects of RNA misexpression due to cis-acting regulatory variants (orange triangle) could be buffered through (1) negative feedback of a gene product onto its RNA expression level as investigated here or (2) through compensatory translation efficiency effects as recently proposed (Artieri & Fraser, 2014; McManus *et al*, 2014).  Allelic differential expression (ADE) was estimated from allele-specific read counts in RNA-sequencing (right column) from a cross (F1 generation, top row) of the yeast strains SK1 (red) and S96 (blue) and compared against ADE from its pool of spores (F2 generation, bottom row).  Cis effects yield to ADE in both the hybrid and the pool of spores (left column). In contrast, local trans effects including feedback only yield to ADE in the pool of spores (center column). Distant trans effects do not yield to ADE neither in the hybrid nor in the pool of spores (being averaged out).

Recently, two studies have assessed the role of translation in buffering variations in RNA expression (Artieri & Fraser, 2014; McManus *et al*, 2014). In both studies, allelic differential expression(ADE) was compared to allelic differential translation efficiency estimated from allele-specific ribosome occupancies in a cross of the yeast species *S. cerevisiae* and *S. paradoxus*. Allelic differential expression indicates effects of cis variants, i.e. regulatory variants that act on one but not on both alleles of a gene (Cowles *et al*, 2002; Yan *et al*, 2002). Focusing on genes with both a significant ADE and significant allele-specific translation efficiency differences, these studies reported an excess of translation efficiency differences opposing to the allelic differential expression. In contrast, Muzzey *et al* (2014) reported a genomewide trend for reinforcing ADE during translation in the yeast *C. albicans*. As these studies used distinct statistical procedures and species, it is hard to compare them and conclude about the generality of these findings. It is appealing to conceive translation as a check point to counter allelic expression imbalance (Fig1A). However, a general mechanism that could sense mRNA allelic imbalance and regulate translation accordingly is hard to imagine. Instead, the most likely explanation for translational buffering is the selection for compensatory mutations (Artieri & Fraser, 2014; McManus *et al*, 2014). Hence, variation in translation efficiency might contribute to buffering but does not appear as an intrinsic mechanism that yields robustness against newly arisen regulatory variants.

Alternatively, Denby *et al* (2012) have proposed that negative feedback controlling the level of RNA expression could be a common mechanism to buffer effects of regulatory variants (Fig 1A). Negative feedback would buffer expression differences by exerting a stronger repression on alleles with higher expression levels and a weaker repression on alleles with lower expression levels. Screening for auto-regulated transcription factors in yeast, Denby *et al* (2012) found *ROX1* to be under strong negative feedback. Mutant experiments showed that this negative feedback confers robustness to the expression of *ROX1* in the face of naturally occurring allelic variants present in a set of divergent yeast strains. This study demonstrated for a single gene that negative feedback could act as a buffering mechanism for regulatory variants. However, data about the extent of feedback mechanisms genomewide and its importance for buffering regulatory variants are still lacking.

Here, we sought to quantify the extent of buffering by feedback against naturally occurring regulatory variants genomewide. To this end, we devised a novel experimental design in which ADE in a hybrid of two yeast strains is compared against ADE in a pool of spores of the same cross (Fig 1B). We distinguish three types of regulatory variants (Rockman & Kruglyak, 2006). First, cis-regulatory variants affect by definition only the allele of the same chromosome and induce ADE in both the hybrid and the pool of spores (Fig1C, left column). Instances of cis-regulatory elements include transcription factor binding sites and regulatory elements in the UTR. Second, local trans mechanisms, which act in trans and are inherited together with the gene they affect, induce ADE in the pool of spores. However, as any trans effect (Cowles *et al*, 2002; Yan *et al*, 2002), local trans mechanisms act in the hybrid unspecifically on both alleles and thus do not induce ADE in the hybrid (Fig 1C, middle column). Local trans regulation can be due to the product of the gene itself (feedback) or to another gene in linkage disequilibrium such as a nearby encoded transcription factor (Ronald *et al*, 2005). Local trans regulation can reduce the ADE in the spores compared to the hybrid, if it counteracts the cis regulation (Fig 1B). Third, distant trans mechanisms, which are encoded on another chromosome or at a distant, unlinked locus of the same chromosome, are inherited independently of their target genes in the spores. Hence, effects of distant trans mechanisms are averaged out across the population of spores and thus do not contribute to ADE (Fig 1C, right column). Altogether, comparison of ADE in the hybrid against the pool of spores thus enables the dissection of local regulation into cis and local trans (including feedback) effects.

We find that buffering through local trans regulation is widespread, typically compensating for 15% of cis-regulatory effects on individual genes. It is stronger for genes with essential function and with low to middle level of expression. In contrast, re-analysis of published ribosome profiling data (Artieri & Fraser, 2014) did not support buffering at the translational level. Altogether, our results indicate that negative feedback plays an important role in buffering regulatory consequences of genetic variants.

## Results

### Dissecting cis- and local trans-regulatory effects

The reference lab strain S96 (Mortimer & Johnston, 1986; Cherry *et al*, 2012) was crossed with the wild isolate SK1 (Kane & Roth, 1974; Nishant *et al*, 2010). Sporulation, germination, and overnight growth of the pool of spores led to the enrichment of alleles due to natural selection as well as technical selection for a single mating type (Ehrenreich *et al*, 2010; Parts *et al*, 2011; Wilkening *et al*, 2014). To control for this bias, allele frequencies were robustly estimated from DNA sequence data of the pools (Materials and Methods). S96 and SK1 are genetically distant strains (0.7% divergence, Nishant *et al* (2010)), allowing investigation of a large set of regulatory polymorphisms and alleles. We identified 7,231 genes of a comprehensive S96 transcriptome annotation (Xu *et al*, 2009) that are common to both backgrounds by reciprocal best alignments with at least an identity of 95% (Materials and Methods). Out of these, the 6,934 (96%) genes that showed expression for both alleles and carried at least one polymorphism were amenable to allele-specific expression profiling by RNA-sequencing (Fig 1B, Materials and Methods).

RNA-sequencing showed high reproducibility between biological replicates, though higher between hybrids than between pools of spores (Supplementary Fig S1, Spearman correlation 0.98 and median coefficient of variation of expression level of 14% in hybrids versus 0.96 and 24% in spores, respectively). Deep sequencing led to 6,691 genes (93%, 5,078 coding and 1,613 non-coding) with more than 10 allele-specific reads on average per sample (median 1,044), for which we considered to have enough data to investigate their allele-specific regulation quantitatively. Cis and local trans effects were estimated using a generalized linear model of allele-specific RNA-sequencing read counts (using the software DESeq2 by Anders & Huber (2010), Materials and Methods). In contrast to standard methods that estimate allelic differential expression from RNA-sequencing data (Bullard *et al*, 2010; Emerson *et al*, 2010; McManus *et al*, 2010), our approach (i) jointly modeled all replicates, avoiding summarizations of per-replicate results that do not take between-replicate variance into account, (ii) modeled over-dispersion of RNA-sequencing read counts, limiting false positive results in comparison with Poisson or binomial models (Anders & Huber, 2010), and (iii) flexibly allowed controlling for covariates with known (genomic allele frequency) or with unknown (replicate, ploidy) effects. Lack of correlation of cis effect estimates with genomic allele frequency (Supplementary Fig S2) and L-shaped distribution of *P*-value (Supplementary Fig S3 center) indicated the validity of the method.

Overall, 984 (15%) genes showed strong and significant cis effects (cis genes, effect > 1.5-fold and FDR < 0.2, Benjamini-Hochberg correction here and in the following) and 54 (1%) genes showed strong and significant local trans effects (effect > 1.5-fold and FDR < 0.2, Supplementary Fig S3, Materials and Methods). When not filtering by effect size, the prevalence of cis effects in this cross (23%, 1,552) was in line with former reports in yeast (∼33%, 1,400 of 4,140 genes in Tirosh *et al* (2009); 19% cis, 830 of 4,282 genes in Emerson *et al* (2010)), fly (18% cis, 1,359 of 7,631 in Suvorov *et al* (2013)), and mice (31% cis, 3,149 of 10,090 genes in Goncalves *et al* (2012)). Local trans genes were enriched for genes encoding proteins that localize in the extracellular region (Gene Ontology enrichment (Ashburner *et al*, 2009), Fisher's test, FDR = 0.02), in agreement with trans effects acting often due to variations in sensory processes (Tirosh *et al*, 2009). Most of the local trans genes do not encode transcription factors (Materials and Methods) in line with the lack of enrichment of transcription factors among trans-acting regulatory loci (Yvert *et al*, 2003) and thus were missed in the previous transcription factor screen (Denby *et al*, 2012). On the other hand, *ROX1* showed no evidence for local trans regulation in our study, most likely because its feedback works under hypoxic conditions (Denby *et al*, 2012). The much smaller amount of genes with significant local trans effects in comparison to the amount of genes with significant cis effects does not prove that local trans effects are less prevalent. Instead, this difference is likely a consequence of the limited statistical power for calling local trans effects, which relies on determining a difference between spore ADE and hybrid ADE. In comparison, there is much higher power to detect cis effects which mainly relies on determining hybrid ADE. Nonetheless, genes under documented feedback regulation including *PHO84* (Wykoff *et al*, 2007) and *AMN1* (Wang *et al*, 2003; Yvert *et al*, 2003; Ronald *et al*, 2005) were identified (Supplementary Fig S4 top). This shows that genuine strong local trans effects could be detected. Moreover, 14 out of the 54 genes showed complete buffering of cis effects through local trans regulation, that is they exhibited a strong ADE in the hybrid and essentially equal allelic expression in the pool of spores (hybrid count ratios larger than 1.5 and spore count ratios smaller than 1.5, examples in Supplementary Fig S4 bottom). Together, these findings indicate that buffering through local trans regulation might be frequent.

### Local trans effects buffer cis effects genomewide

**Figure 2:**
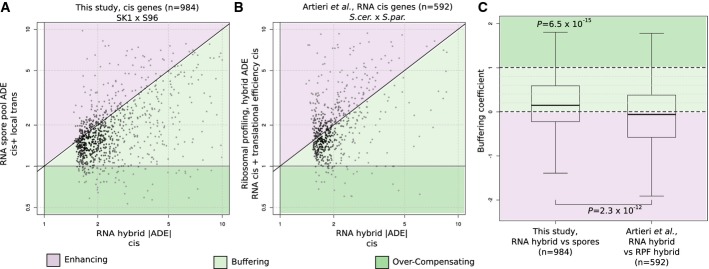
Local trans effects, but not translation, buffer ADE  Scatter plot of allele-specific expression ratios in the pool of spores (*y*-axis) against hybrid (*x*-axis) for the genes with cis effect (984 cis genes). For both axes and on a gene basis, the allele with the lower expression level in the hybrid is taken as reference (denominator). ADE in the hybrid measures cis-regulatory effects (*x*-axis). Three categories of genes are distinguished depending on the resulting ADE in the pool of spores (*y*-axis, due to cis and local trans regulation): compensated (dark green background) with canceled or opposite ADE (over compensation), buffered (light green) with reduced ADE, and enhanced ADE (purple). Most of the genes are buffered.  Analogous to (A) but for the 592 RNA cis genes of the Artieri & Fraser (2014) dataset. Ribosomal profiling ratios (*y*-axis) of a cross between *S. cerevisiae* and *S. paradoxus* are compared against RNA ratios (*x*-axis) of the same hybrid. The mass of the data lies at the diagonal indicating that RNA cis effects in the hybrid are not buffered translationally.  Quartiles (boxes) and 1.5 times the interquartile range (whiskers) of the buffering coefficient for the gene sets from (A), left and (B), right. The buffering coefficients at RNA level are significantly greater than zero (left, median = 0.147, *P* < 6.5 × 10^−15^, one-sided Wilcoxon test), whereas they are not at translational level (right). Both distributions differ significantly (*P* = 2.3 × 10^−12^, two-sided Wilcoxon test).

As statistical power on individual genes is limited, we also analyzed local trans regulation genomewide. In this experimental setup, buffering can only be assessed for genes showing a cis effect in the first place. For the 984 cis genes, allelic expression imbalances typically agreed in direction, but were weaker for the pool of spores compared to the hybrid (Fig2A, mass of the data subdiagonal). To quantify the amount of buffering of cis effects, we defined the buffering coefficient *C* as one minus the log-ratio of allele-specific expression in the spores versus the hybrid (see Materials and Methods for definition and unbiased estimation). The buffering coefficient has a value of 0 in the absence of buffering (equal ADE in the pool of spore and hybrid), 1 for complete compensation (ADE in the hybrid but no ADE in the pool of spores). The buffering coefficient is greater than 1 in case of over-compensation and is negative if local trans effects enhance cis effects. More than half of the genes with cis effects showed at least partial buffering (60% with *C* above 0). Local trans buffering appeared to affect all classes of genes, since no gene ontology category was significantly enriched (Fisher's test, FDR < 0.1). Moreover, no significant association was found between buffering coefficient and gene features that have been associated with gene expression variability (TATA box) or dosage compensation in fly (gene length) (Supplementary Fig S5). The trend for buffering was robust to the definition of cis genes as it was still detectable across all genes (Supplementary Fig S6A). Hence, genomewide cis effects tend to be partially buffered by local trans-regulatory mechanisms. These local trans mechanisms buffer typically 15% (Fig 2C, median *C* = 0.148; 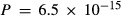, one-sided Wilcoxon test) of allelic expression log-ratios caused by cis-regulatory variants (Fig 1A).

To compare the amount of buffering by local trans mechanisms against buffering by translation efficiency, we re-analyzed one ribosome-profiling dataset (Artieri & Fraser, 2014) following the same statistical procedure as above. Here, the ribosome profiles of the hybrid substitute for the transcription profiles in the pool of spores (Materials and Methods). A total of 592 genes were identified as having cis differences on RNA expression (effect > 1.5-fold and FDR < 0.2). For these genes, allelic differential levels of ribosome-bound RNAs had typically the same extent as allelic differential levels of expression of the RNAs in the hybrid (2Fig B, mass of the data along the diagonal; Fig 2C, median buffering coefficient −0.058, 54% with *C* < 0). This observation was robust with respect to the definition of cis genes, since no support for translation efficiency buffering was detectable across all genes, too (Supplementary Fig S6B). We did not find an enrichment for translation efficiency opposite to ADE either when we focused on genes with both a significant ADE and significant allele-specific translation efficiency differences as the original study did (164, 54%, genes out of 303 genes with FDR < 0.2 for both effects had opposing ADE and translation efficiency, *P* = 0.17 two-sided Binomial test). Both previous publications (Artieri & Fraser, 2014; McManus *et al*, 2014) could have been misled by the fact that translation efficiency estimates were technically anti-correlated with RNA level estimates (Albert *et al*, 2014) and by the fact that the measurement variance was larger than assumed (Supplementary Information).

### Local trans buffering is stronger for essential genes

**Figure 3:**
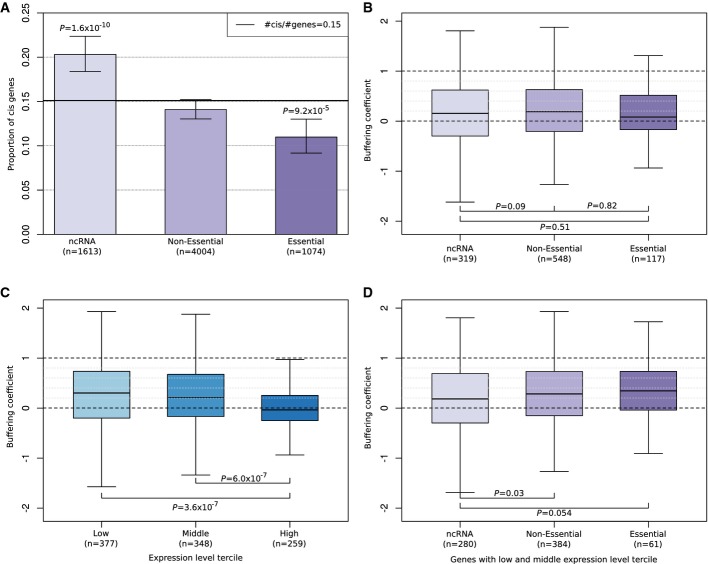
Local trans buffering is stronger for genes important for fitness and with low to middle levels of expression  Proportion of cis genes by gene category. Essential genes show a lower cis gene proportion than genomewide (horizontal line), whereas non-coding RNAs are enriched for cis genes (*P*-value from two-sided Fisher's test, error bars indicate 95% confidence intervals for binomial proportions).  Quartiles (boxes) and 1.5 times the interquartile range of the buffering coefficient for cis genes grouped by gene category. No significant differences detectable. *P*-values are computed with an one-sided Wilcoxon test with the alternative hypothesis that essential genes are more buffered than ncRNA, analogously for non-essential.  Analog to (B) but for cis genes grouped by expression level tercile. Highly expressed genes are less buffered than genes with low and middle expression levels. *P*-values are computed with a two-sided Wilcoxon test.  Same as (B) but for cis genes only at low and middle expression levels. At these levels of expression, buffering positively associates with fitness relevance category.

If local trans regulation confers robustness against regulatory variants, then one would expect it to be stronger at genes important for fitness. We tested this hypothesis by classifying genes into three categories with increasing fitness relevance: 1,613 non-coding genes (24%, ncRNA), 4,004 non-essential protein-coding genes (60%, non-essential), and 1,074 essential protein-coding genes (16%, essential). The proportion of cis genes in each category was inversely related to fitness relevance (Fig3A), whereby ncRNAs were enriched for cis genes (20%, 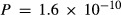, Fisher's test) and essential genes were depleted for cis genes (11%, 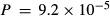, Fisher's test). This result also held when controlling for expression level and considering the combination of two FDR thresholds (0.1 and 0.2), with and without fold change cutoff (Supplementary Fig S7). The association of cis effects with gene categories is in line with former reports limited to protein-coding genes (Tirosh *et al*, 2009; Emerson *et al*, 2010) and consistent with the idea that selection on regulatory elements is more important for coding than non-coding genes and for essential than non-essential genes. Surprisingly, the buffering coefficient and fitness relevance did not correlate (Fig3B). However, stratifying genes into three equally large groups with low, middle and high average expression levels revealed that highly expressed genes showed lower buffering coefficients compared to the two other groups (Fig3C, median buffering coefficient = −0.036 versus 0.284 and 0.202 with 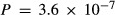 and 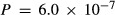 for low and middle levels, respectively. Wilcoxon test, Materials and Methods, Supplementary Fig S8 top). This result held when considering combinations of FDR and fold change cutoffs as above (Supplementary Fig S9). A plausible explanation for this observation is that buffering is less needed for highly expressed genes because RNAs are produced in excess and thus variation in their expression level has less phenotypic impact. Consistent with this hypothesis, the buffering coefficient was found to be positively associated with fitness relevance when restricted to genes with low and middle levels of expression (Fig 3D, Supplementary Fig S8 bottom). These results provide clear evidence for two regulatory strategies conferring robustness against regulatory variants: excess amount of RNA on the one hand, and buffering through local trans regulation for low to middle levels of expression on the other hand.

### Local trans buffering is primarily due to negative feedback

**Figure 4:**
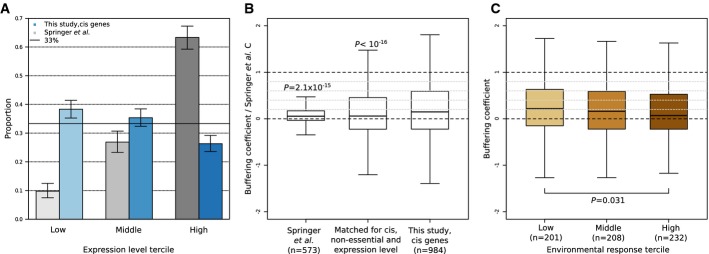
Local trans buffering is primarily due to negative feedback  Proportion of expression levels in Springer *et al* (2010) dataset (gray) and from cis genes in this study (blue). Due to technical limitations, Springer and colleagues’ dataset is enriched for genes with high levels of expression. Error bars indicate 95% confidence intervals for binomial proportions.  Quartiles (boxes) and 1.5 times the interquartile range (whiskers) of Springer and colleagues’ *C* coefficient (left), of the buffering coefficient estimated in this study for cis genes with expression level distribution and gene category matching Springer and colleagues’ dataset (Materials and Methods, center), and of the buffering coefficient estimated in this study for all cis genes (right). Springer and colleagues’ *C* mathematically corresponds to the here defined buffering coefficient under simple assumptions (Supplementary Information). Significant buffering is found in Springer's gene set (*P* = 2.1 × 10^−15^, one-sided Wilcoxon test). The significantly lower amount of buffering (left, median = 0.055) compared to the genomewide amount of buffering reported here (right, median = 0.148) is explained by the bias for non-essential and highly expressed genes in Springer and colleagues’ experimental setup (median = 0.058 for matched gene properties, center).  Quartiles (boxes) and 1.5 times the interquartile range of the buffering coefficient for cis genes (*y*-axis) by tercile of median absolute value of gene expression log2-ratio in response to more than 1,500 environmental changes (Tirosh *et al*, 2009; *x*-axis). Environmental expression data were available for coding genes only (*P*-value one-sided Wilcoxon test).

Buffering by local trans regulation can be caused by the gene itself (negative feedback) or by any other gene in linkage disequilibrium with it. Although negative feedback provides a simpler explanation for our data since the buffering is accomplished without the need for compensatory mutations, both mechanisms could be at play. To understand which of these two mechanisms is the major contributor to buffering, we revisited data of a previous study in which protein levels of 730 genes in diploid strains with one gene copy deleted were compared to wild-type levels (Springer *et al*, 2010). In this experiment, compensatory mutations had no time to occur since the deletion was introduced artificially. Consequently, only the effect of feedback was measured. Springer and colleagues’ screen was technically limited to non-essential genes and to genes with high levels of expression (63% in the highly expressed tercile, Fig4A), that is for two gene categories for which we detected lower amounts of buffering than genomewide. Nonetheless, we found evidence for buffering in this dataset (Fig4B; median *C* = 0.055, 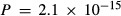 for Springer *et al* (2010), one-sided Wilcoxon-test). Moreover, buffering in these data was comparable to the amount of local trans buffering we observed for genes with matched properties (Fig 4B, median *C* = 0.058, Materials and Methods and Supplementary Information). Hence, these deletion experiments indicate that negative feedback is the primary mechanism for local trans buffering. A further feature distinguishing negative feedback from compensatory mutation is that negative feedback also confers robustness to environmental variations. Consistently, the buffering coefficient of the cis genes negatively associated with expression response to more than 1,500 environmental perturbations (Tirosh *et al*, 2009) (median buffering coefficient = 0.22 for the low versus 0.07 for the high tercile of environmental response, *P*-value = 0.031, one-sided Wilcoxon test, Fig 4C, Supplementary Fig S10). Altogether, these results indicate that local trans buffering is primarily due to negative feedback rather than due to compensatory mutations.

## Discussion

We found that compensatory local trans-regulatory mechanisms buffer typically 15% of RNA level log-ratios caused by naturally occurring cis-regulatory variants in *S. cerevisiae*. Local trans mechanisms involve the gene itself (feedback) or trans-acting variants in its genetic vicinity. Analysis of expression data of heterozygous deletions indicates that this buffering is primarily due to negative feedback regulation and not due to compensatory mutations. In addition, we did not find evidence for translational buffering to be common when reanalyzing ribosome profiling data of a cross between two yeast species, even though translational buffering occurs for specific instances. The intensity of buffering through local trans regulation was lower for highly expressed genes, suggesting that the sheer amount of transcripts available for these genes confer robustness against cis-regulatory variants. In low to middle range of expression, buffering was increasing across the three categories, non-coding, non-essential coding, and essential coding genes, correlating with presumed functional importance.

We dissected local regulation into its cis and trans components using a novel experimental design in which ADE in a yeast hybrid strain was compared against ADE in a pool of its spores. In contrast, former dissection of local regulation was performed in two steps (Ronald *et al*, 2005). First, polymorphisms in the vicinity of genes that significantly associated with their expression across a population of spores were identified (eQTL mapping). Second, the estimated effect of these local eQTLs was compared to allelic differential expression in a hybrid strain. The advantage of our experimental design is first economic, because the spores are pooled whereas eQTL mapping requires typically dozens of individual spores to be transcription profiled. Second, our design suffers less from confounders such as batch effects that can give false associations in eQTL mapping. Third, ADE in the hybrid is more comparable to ADE in the pool of spores than to eQTL effects because in the former case the same experimental protocol and the same analysis are applied. One should note that amplification and sequencing biases could favor one allele thereby leading to overestimated ADE. However, the same bias applies similarly to the pool and to the hybrid and thus does not affect our observation that ADE is lower in the pool than in the hybrid. Our experimental design could be applied to study other levels of gene regulation where local trans mechanisms, and in particular regulatory feedback, could play a significant role, including synthesis and decay of RNA, translation, and protein levels (Khan *et al*, 2012).

Our findings have implications for the understanding of dosage compensation, that is the buffering of expression level in case of gene copy number variation. Unlike for sex chromosomes, the prevalence and the mechanisms for dosage compensation on autosomes are poorly understood. Buffering in the 10–20% range was reported for a set of seven autosomal single copy deletions in fruit fly (Lundberg *et al*, 2012). In contrast, Springer *et al* (2010) reported a general lack of dosage compensation in yeast. Our study shows that these observations are more in agreement with each other than they seem to be. We found that buffering against cis-acting regulatory variants in yeast is typically of 15% genomewide, and that Springer and colleagues’ heterozygous deletion screen was biased for genes with little buffering (about 5%). Hence, the extent of buffering appears to be conserved from yeast to fly. Moreover, we found that buffering is primarily due to negative feedback which confers robustness against single nucleotide polymorphisms and short indels as well, as supported by the fact that we assessed genes with more than 95% identity between the two parental strains. Together, these results suggest that dosage compensation of autosomal genes in higher eukaryotes might be explained to a large extent by negative feedback, that is by a mechanism that generally buffers regulatory variants rather than by a copy number surveillance pathway.

In 1942, Waddington hypothesized the existence of buffering mechanisms against genetic variants that would explain the remarkable stability of developmental processes among individuals (Waddington, 1942; Flatt, 2005). It is still unclear to date, which buffering mechanisms act across the stages of phenotypic expression, from DNA to RNA, protein and cellular phenotypes, and what their respective contribution is. Robustness against coding variations can be explained by redundancy, such as diploidy, copy number variation, and functional duplication (Hartwell *et al*, 1999; Hartman *et al*, 2001). Our data show that already at the level of RNA expression, buffering is widespread. We could estimate its effect and identified negative feedback as the predominant mechanism. Protein abundance of orthologous genes has been shown to be more conserved than mRNA abundance across all domains of life ranging from bacteria to fungi and primates (Schrimpf *et al*, 2009; Laurent *et al*, 2010; Khan *et al*, 2013). Thus, further mechanisms buffering regulatory variants downstream of RNA expression remain to be identified (Dahan *et al*, 2011; Vogel, 2013). One possibility is that negative feedback is also common for controlling protein levels.

Buffering plays an important role in evolution because it confers robustness to mutations on the one hand and allows the accumulation of cryptic genetic variants in the population that might give selective advantage under new environmental conditions on the other hand. In this context, a capacitor is a switch capable of releasing previously cryptic heritable variation (Masel & Siegal, 2009). Since feedback loops themselves can be impaired, through mutations as in the case of *ROX1* or environmental changes, we suggest that negative feedback loops could function as capacitors.

## Materials and Methods

### Data availability

All raw sequencing files for DNA and RNA samples, processed DNA coverage as well as raw read counts per transcript and sample are available at gene expression omnibus (GEO id: GSE61553). Supplementary Table S1 contains raw expression counts for filtered genes, normalized counts, results of the statistical analysis and further annotation used to produce the figures. Supplementary Table S2 contains the raw read counts per transcript and sample shared by Carlo Artieri and Hunter Fraser (personal communication). Supplementary Table S3 contains raw expression counts for filtered genes, normalized counts, results of the statistical analysis of the ribosomal data based on Supplementary Table S2 (Artieri & Fraser, 2014).

### Yeast strains

In this study we used the hybrid strains and the pools of spores used for bulk segregant analysis from a recent QTL study by Wilkening *et al* (2014). Strains were grown in YPD medium (1% yeast extract, 2% peptone and 2% glucose).

### DNA sequencing

DNA sequencing data from Wilkening *et al* (2014) were used to estimate allele frequencies for our hybrid and spore samples. Note that DNA fragmentation was done with a Bandelin and a Covaris sonicator, except for spore pool B, where only a Covaris fragmentation was applied, which led to reduced coverage.

### Transcriptome profiling

Total RNA was isolated by a standard hot phenol method followed by DNase treatment using Turbo DNA-free kit (Ambion). Strand-specific total RNA-Seq libraries were prepared as described in Wilkening *et al* (2013) which is a modified protocol of Parkhomchuk *et al* (2009). Briefly, 10 μg of total RNA was fragmented by incubating the samples at 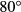
C for 5 min in the presence of RNA fragmentation buffer (40 mM Tris-acetate, pH 8.1, 100 mM KOAc and 30 mM MgOAc). The fragmented RNA was purified using 1.5× Ampure XP Beads (Beckman Coulter Genomics). Eluted RNA was reverse transcribed with 3 μl oligo dT18 with a VN anchor (1 μM, Invitrogen), 3 μl random hexamers (30 ng/μl, Invitrogen) and 2 μl   10 mM dNTPs. The samples were incubated at 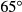
C for 5 min and transferred to ice. Of 8 μl 5× First strand buffer (Invitrogen), 4 μl DTT 0.1 M, 0.5 μl actinomycin D (1.25 mg/ml) and 0.5 μl RNasin plus RNase inhibitor (Promega) were added to each sample and the samples were then incubated at 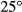
C for 2 min. Following this, 0.5 μl Superscript III reverse transcriptase (200 U/μl, Invitrogen) was added. The retrotranscription was carried out at 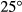
C for 10 min, and at 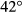
C for 50 min, and inactivated at 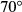
C for 15 min. After cleanup, the 2^nd^ strand cDNA synthesis was done with dUTPs instead of dTTPs. For ligation, 1 μl of forked paired end multiplexed adaptors (40 μM) was used. The dUTPs of the second strand were hydrolyzed by incubating the samples at 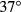
C for 30 min with 5 units of UDG in UDG reaction buffer (NEB). The samples were purified using 1× Ampure XP beads. After 10 cycles of PCR amplification and cleanup, samples were submitted to the EMBL core facility for 100-bp paired end sequencing on a HiSeq 2000 (Illumina).

We produced 186, 148, 204, and 188 million read pairs of good quality (R bioconductor package ShortRead, quality score of more than 30) for hybrid A, hybrid B, spore A, and spore B, respectively.

### Genotyping and allele frequencies

S96 is isogenic to S288c besides the mating type, and therefore, we could use the reference genome of the *S. cerevisiae* database (Cherry *et al*, 2012). We used the allele frequencies computed earlier by (Cherry *et al*, 2012). The coverage of the spore pool B DNA sample was lower than for the other three samples (see DNA sequencing section); hence, we have allele frequencies for about 60,000 and 10,000 SNPs, respectively. To adjust the SNP coordinates, we lifted them from S288c version R63 to R64. We smoothed the allele frequencies over a window of 28,000 bp (∼10 centimorgan) using local binomial likelihood estimation (R CRAN package locfit). We observed a mapping bias toward the S288c genome (median S288c allele frequency 0.52), most likely due to the better annotated reference genome. This artificial bias was used to correct the spore frequency estimations. Those mapping-bias-corrected spore allele frequencies were used to correct the read counts for the statistical model. A similar mapping issue was not observed for the hybrid RNA counts.

### Gene annotation

To include also recent non-coding RNAs we used the gene annotation of Xu *et al* (2009) for gene coordinates in the S96 strain (isogenic to S288c). The SK1 gene annotation was generated via bidirectional best hits: Using the coordinates from Xu and colleagues, we extracted the S96 gene sequences from the S288c genome version R64 of the Saccharomyces Genome Database (Cherry *et al*, 2012). These sequences were searched in the SK1 genome using BLAST (Altschul *et al*, 1990) with default parameters. The best hit of this first search became query of the second search in the S96 genome. If this second search resulted in the query of the first, we considered the gene pair as ortholog candidates. Every pair with an alignment identity of more than 95% was considered orthologous. This includes also longer indels and does not restrict our analysis to single nucleotide variants.

Additionally, expression levels for each gene are defined as the average read counts divided by the mean gene length over both strains. These levels were sorted and categorized into three equally sized groups: *Low*, *Middle* and *High* using *cut2* (R package Hmisc). Transcription factor annotation was taken from MacIsaac *et al* (2006).

### Mapping and read counts

RNA-seq reads were mapped to the genomes of S96 and SK1 jointly. GSNAP (Wu & Nacu, 2010) was used allowing for four mismatches with novel splice site detection enabled, apart from that we used default parameters. We classified mapped read pairs into three categories: common, only SK1, and only S96. Common reads matched equally well to both genomes and therefore are not apt to measure ADE. Only the strain-specific and proper-paired alignments can led to ADE and were filtered by their SAM flags (i.e. 83/163 and 99/147) for our statistical model. Additionally, if one read had one proper pair and one mate aligned to the same chromosome on the other allele, it was considered as specific, too. All other reads were discarded together with the common reads.

The filtered alignments were processed with *htseq-count* (Anders *et al*, 2014) using *intersection-strict* as overlap mode to generate read counts per gene. *Strict* means that a read or read pair has to align completely inside the annotated gene region to be counted. As gene annotation we used our expressed orthologs with start and end extended by 50 bp to increase sensitivity.

### Statistical modeling

The raw counts of reads (integer values) per annotated gene are prone to systematic biases that need to be corrected. During the growth of the spores artificial (one mating type) and natural selection takes place (Ehrenreich *et al*, 2010; Parts *et al*, 2011). To deal with this bias, we used the genomic allele frequencies of the spores for correction (Supplementary Fig S2, see genotyping and allele frequencies section). Additionally, we corrected for length differences between the strains genewise as well as the standard sample size factors by DESeq2 (Anders & Huber, 2010). Furthermore, we modeled additional confounding factors for diploid cells, and the biological replicate of each hybrid and spore pool (design matrix, Table1). Hence, allele-specific read counts 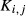 were modeled according to the following generalized linear model: 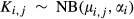
1

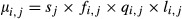
2

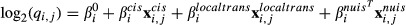
3where NB is the negative binomial distribution, 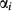 is a gene-specific dispersion parameter; 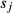 is the size factor of sample *j*; 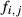 is the allele frequency of gene *i* in sample *j*; 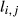 is the length of the allele for gene *i* in sample *j*. The value of 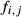 is 0.5 in the hybrid sample and is robustly estimated from genomic DNA sequencing in the pool. 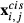 is 1 for allele K and 0 otherwise. 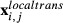 is 1 in the pool for allele K and 0 otherwise. 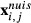 represents all nuisance parameters to control for: *diploid*, *hybrid B*, *pool B* (Table1). The model was implemented with the R/Bioconductor package DESeq2 (Anders & Huber, 2010), which provides robust estimation of the size factors and of the dispersion parameters.

**Table 1:** DESeq design matrix. A cell denotes whether we can observe an effect of the modeled factor (column) in the specified sample (row). Samples split by strain and biological replicate

Sample/Factor	Cis	Local trans	Diploid	Hybrid B	Spore B
Hybrid A only SK1	1	1	1	0	0
Hybrid A only S96	0	1	1	0	0
Hybrid B only SK1	1	1	1	1	0
Hybrid B only S96	0	1	1	1	0
Spore A only SK1	1	1	0	0	0
Spore A only S96	0	0	0	0	0
Spore B only SK1	1	1	0	0	1
Spore B only S96	0	0	0	0	1

After the correction and fitting process we removed genes from further analysis that had less than ten reads average count over all samples, in order to increase our detection power at the same type I error (Supplementary Fig S3 top row; Anders & Huber, 2010; Bourgon *et al*, 2010). This minimal expression filtering resulted in 6,691 genes. Accordingly, we corrected the *P*-values for multiple testing using false discovery rate (Benjamini & Hochberg, 1995). Supplementary Table S1 provides normalized counts together with fitted coefficients and further gene annotation.

### Analysis of ribosome profiling data

We re-analyzed read count data kindly provided by Carlo Artieri and Hunter Fraser (personal communication, Supplementary Table S2), adopting our model to the hybrid data from RNA-seq and ribosomal profiling. The ribosome-bound fraction was assumed to be the product of the expression level and the binding affinity to RNA, a proxy for translation efficiency (Ingolia *et al*, 2009). Accordingly, allele-specific read counts 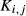 were modeled according to the following generalized linear model: 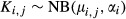
4

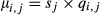
5

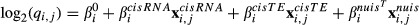
6where NB is the negative binomial distribution, 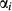 is a gene-specific dispersion parameter; 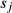 is the size factor of sample *j*; 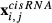 is 1 for the *S. paradoxus* allele and 0 otherwise. 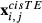 is 1 in the ribosome-bound fraction for the *S. paradoxus* allele and 0 otherwise. 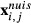 represents nuisance parameters that were controlled for: baseline translation efficiency and overall replicate effect (Table2). The model was implemented with the R/Bioconductor package DESeq2 (Anders & Huber, 2010). Supplementary Table S3 provides normalized counts together with fitted coefficients and further gene annotation.

**Table 2:** DESeq design matrix for ribosome profiling data. Value of covariates by sample for the Equation 4

Sample	RNA cis	TE cis	RNA bias	Hybrid rep2
Hybrid RNA 1 SCER	1	0	1	0
Hybrid RNA 2 SCER	1	0	1	1
Hybrid RNA 1 SPAR	0	0	1	0
Hybrid RNA 2 SPAR	0	0	1	1
Hybrid RIBO 1 SCER	1	1	0	0
Hybrid RIBO 2 SCER	1	1	0	1
Hybrid RIBO 1 SPAR	0	0	0	0
Hybrid RIBO 2 SPAR	0	0	0	1

### Buffering coefficient

#### Definition

To quantitatively estimate how much cis effects are buffered by local trans effects, we defined the buffering coefficient *C* as: 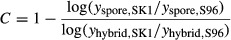
7where *y* denotes the RNA expression level.

In order to estimate buffering at the transcriptional level, we also defined buffering coefficient when comparing ribosome profiling data (RP) and RNA levels in the *S. par*. × *S. cer*. cross. 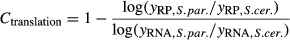
8where 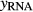 denotes the RNA expression level, and 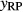 the ribosome occupancy.

Note that both for the local trans regulation case and for the translation efficiency case, *C* is ill-defined for hybrid RNA ratios close to zero. This is equivalent to say that buffering can only be assessed if there is a cis effect in the first place. We therefore restricted the analysis of buffering for genes with significant and sufficiently large cis effects.

#### Calibration

We defined as raw buffering coefficient the quantity: 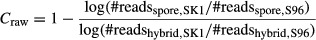
9


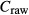
 is a biased estimator of the buffering coefficient *C* defined by Equation 7. We empirically derived an unbiased estimator of *C* by inferring the relationship between 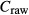 and *C* from simulations for all values of *C* in [0.0.5] with a 0.005 spacing. For each simulated value of *C*, read counts for every gene *i* were simulated by random draws according to Equations 1,2,3, keeping all the parameters fixed to their estimated values on the primary dataset, except for substituting 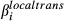 with 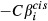. On these simulated genomewide read counts, the exact same analysis as for the primary dataset was performed (i.e., including filter for minimum read counts, DESeq2 normalization and fits, and filter for large and significant cis effects) and the median 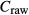 across cis genes was computed. To obtain an unbiased estimator of translational buffering for the ribosome dataset, the same procedure was applied substituting 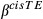 with 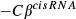. For both datasets, we observed a linear relationship between the simulated true *C* and the median 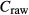 (Supplementary Fig S12A and B, Pearson correlation  > 0.99) and used the linear regression fit as calibration function. This linear transformation of 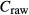 was then used for all further analysis as buffering coefficient *C*.

#### Significance

To assess the significance of the median buffering coefficient, data were simulated under the null hypothesis of independence between cis effects and local trans effects in a semi-parametric fashion. A total of *B* = 1,000 bootstrap genomewide datasets were generated by permuting the estimated local trans effects 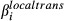 between genes while keeping all remaining parameters fixed to their estimated values on the primary dataset and drawing counts according to Equations 1,2,3. On these simulated genomewide read counts, the exact same analysis as for the primary dataset was performed (i.e., including filter for minimum read counts, DESeq2 normalization and fits, and filter for large and significant cis effects) and the median buffering coefficient across cis genes was computed.

One-sided *P*-value was then estimated by (Davison & Hinkley, 1997): 
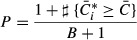
where 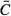 is the median buffering coefficient in the observed dataset and 
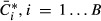
 are the bootstrap values of the median buffering coefficient (Supplementary Fig S12C). The same procedure was applied to the ribosome dataset whereby the estimated translation efficiency estimates 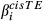 were permuted across genes (Supplementary Fig S12D).

#### Comparison with Springer's C

Comparison with Springer *et al* (2010) data was done for the same growth medium as the one used in this study (rich growth medium YPD). Distribution of our buffering coefficient under matching distribution of gene category and expression levels (Fig 4A, central box) was obtained by (i) restricting to non-essential genes and (ii) randomly samplinzg 1,000 times with replacement the same number of genes in each tercile of expression as in Springer and colleagues’ database.

## Supporting Information

Supplementary Table S3

Review Process File
