# Supplementary material for: Negative feedback buffers effects of regulatory variants
Source: Mol Syst Biol. 2015 Jan 29;11(1):785. doi: 10.15252/msb.20145844 (PMC4332157; doi:10.15252/msb.20145844)
Supplement: Supplementary file 1 [file msb0011-0785-sd1.pdf]

Supplementary information: Negative feedback buffers effects of regulatory variants

Daniel M. Bader[1], Stefan Wilkening[2], Gen Lin[2], Manu Tekkedil[2], Kim Dietrich[1]
Lars M. Steinmetz[2,3,4] and Julien Gagneur[1,*]

January 8, 2015

1. Computational Genomics, Gene Center, Ludwig Maximilians University, 81377 Munich, Germany.
2. European Molecular Biology Laboratory, Genome Biology Unit, 69117 Heidelberg, Germany.
3. Stanford Genome Technology Center, Palo Alto, California 94304.
4. Department of Genetics, Stanford University School of Medicine, Stanford, California 94305.

* Corresponding Author: Julien Gagneur, gagneur@genzentrum.lmu.de, Feodor-Lynen Strasse 25, 81377 Munich, Germany

Subject categories: System Genetics, Bioinformatics, Gene Expression, Gene Regulation

Keywords: Feedback, Cis regulation, Trans regulation, Canalization, Buffering

Standfirst text and highlights: Local trans regulation, mainly due to negative feedback, buffers effects of cis-regulatory variants by about 15%. This buffering is stronger for essential genes and genes with low to middle expression levels, for which tight regulation matters most.

- Novel experimental design using expression of a diploid hybrid and its haploid spores allows systematic dissection of cis and local trans regulation
- Local trans effects buffer effects of cis-regulatory variants in yeast by typically 15%
- Local trans buffering is primarily due to negative feedback
- Negative feedback as robustness strategy for genes with low to medium expression level

Supplementary text

Improvements on the original analyses of ribosomal profiling data

We improved the assessment of translational buffering compared to the original studies (Artieri and Fraser, 2014; McManus *et al*, 2014) in the following three aspects:

1. **Modeling the biological variance.** In the two original studies, tests for allelic differential expression were performed for each biological replicate separately. One of these tests is a binomial test (McManus *et al*, 2010) and the other one is a more conservative test controlling for differences in mappability and nucleotide content (Bullard *et al*, 2010). To call significant effects over the two biological replicates, the largest *P*-value of the two samples had to be smaller than a threshold and allelic expression imbalance had to agree in direction. Hence, both of these approaches assess the within-sample significance but do not assess the significance of allelic expression ratios compared to the variability of expression levels between biological replicates. We found that allelic expression ratios for genes called significant according to these procedures often had low fold-changes in comparison to the median biological standard deviation (20.2% less than 1.96 times the median standard deviation at a nominal *P*-value of 0.05 for Artieri and Fraser (2014), Supplementary Fig S11), indicating that the extent of many reported effects did not significantly replicate between biological replicates. As comparison, assuming known variance, Gaussian distribution, and same sample size ($n = 2$), a nominal *P*-value of 0.05 is reached for differences of about 1.96 or more standard deviations. With our test, which models both the so-called shot noise (Poisson noise dominating low counts) and the biological noise (dominating the high counts), only 3.1% of the called genes at a nominal *P*-value of 0.05 show less than 1.96 times the median standard deviation (Supplementary Fig S11). Consequently, *P*-values were underestimated with the original statistical tests leading to an abundant fraction of rejected null hypotheses. The same issue affected the significance assessment of translation efficencies.

2. **Independent estimates.** Both studies estimated translational efficiencies as the ratio of RNA levels in the ribosome-bound fraction divided by the RNA expression level. Hence, estimates for translational efficiencies and for expression levels were not independent. Specifically, noise in RNA expression level measurements induce anticorrelation between translation efficiency estimates and expression level estimates. A scatterplot of allelic log-ratios of translation efficiencies versus allelic log-ratios of RNA levels gave the misleading impression that the two quantities are biologically anticorrelated (Fig 3B in McManus *et al* (2014) and Fig 2A in Artieri and Fraser (2014)). In contrast, scatterplot of the untransformed data does not indicate a trend for translational buffering (mass of the data above diagonal, Fig S6B). Because the original statistical tests did not assess the between-replicate variability, most of the effects that were called significant for allelic differences in expression and in translation efficiency were likely due to random variations. Estimated allelic ratio of expression and translation efficiencies of these genes therefore tended to suffer from the anti-correlation and thus to spuriously show opposite effects. Re-analysing the data of Artieri and Fraser (2014) with our test and with filtering criteria matching those of the original analysis (FDR=0.05 and no cut-off on fold change), we found much fewer instances (99) significant for both translation efficiency and cis effects. Among these 99 genes, only 55 (56%) show opposing effects which is not statistically significant ($P = 0.31$, two-sided binomial test).

3. **Considering noise in explanatory variable.** In one of the two original studies, genome-wide trend for compensation at the translational level was estimated by a regression of allelic ratios in the ribosome-bound fraction over the allelic expression ratios (Artieri and Fraser, 2014). An important assumption of linear regression is that there is no noise in the explanatory variable. This was not the case here because the RNA levels are noisy estimates. Linear regression in case of noise in the explanatory variable is known to underestimate the slope (regression to the mean effect), which had led to underestimation of the trend. Compare Fig 2B in (Artieri2014) with supplementary figure S6, here we are instead using principal component analysis.

The two latter points were also noticed by Albert *et al* (2014).

Buffering coefficient *C*

Here we define a measure to quantify the amount of buffering on gene expression. We show that under some assumptions our measure is the same than the compensation metric C of Springer *et al* (2010).

We write a gene expression level y as :

$$y = \alpha^{1-C}\beta^{C} \tag{1}$$

where C is the coefficient of compensation, α is the expression level that the gene would reach in the absence of compensation (i.e. if $C = 0$), β is the expression level that would be reached under full compensation ($C = 1$).

Estimation of *C* in this study

We assume the unlogged expression level of an allele to be the product of cis and trans effects: $y = \text{cis} \times \text{trans}$. Moreover, we assume the cis effect to be independent of the compensation C. Thus the allele expression ratio in the hybrid is independent of C and is the same as in absence of compensation:

$$\frac{y_B^{\text{HYBRID}}}{y_A^{\text{HYBRID}}} = \frac{\text{cis}_B}{\text{cis}_A} = \frac{\alpha_B}{\alpha_A}$$

In the spores carrying allele A or allele B, respectively, the level of expressions are:

$$y_A^{\text{SPORE}} = \alpha_A^{1-C}\beta^C$$
$$y_B^{\text{SPORE}} = \alpha_B^{1-C}\beta^C$$

Hence the allelic expression ratio in the pool of spores is:

$$\frac{y_B^{\text{SPORE}}}{y_A^{\text{SPORE}}} = \left(\frac{\alpha_B}{\alpha_A}\right)^{1-C} = \left(\frac{y_B^{\text{HYBRID}}}{y_A^{\text{HYBRID}}}\right)^{1-C}$$

We therefore use as working definition of the coefficient of compensation C in this study:

$$C = 1 - \frac{\log_2(y_B^{\text{SPORE}}/y_A^{\text{SPORE}})}{\log_2(y_B^{\text{HYBRID}}/y_A^{\text{HYBRID}})}$$

Equivalence with Springer's *C*

Springer and colleagues (Springer *et al*, 2010) assess buffering of a protein's expression using diploid strains in which one of the two alleles is marked by GFP. In the so-called wild type strain, the unmarked allele is kept intact whereas in the heterozygote strain the unmarked allele is deleted. Springer's compensation metric is defined as the $\log_2$-ratio of the GFP expression in the heterozygote over the wild type strain.

We index with A the unmarked allele's constants and with B the GFP-tagged allele's constants. In this experiment, the expression levels of the two alleles are assumed to be the same in absence of compensation (i.e. $\alpha_A = \alpha_B := \alpha/2$) and in presence of compensation ($y_A = y_B$). The feedback acts on the overall expression level $y := y_A + y_B = 2y_B$.

According to Equation 1, we expect the GFP expression level y_B^{WT} to be:

$$y_B^{\text{WT}} = \frac{1}{2}y = \frac{1}{2}\alpha^{1-C}\beta^C$$

The GFP expression level in the heterozygote strain, for which $y_A = 0$, is:

$$y_B^{\text{HET}} = y = (\alpha/2)^{1-C}\beta^C$$

Hence we get:

$$\log_2\left(\frac{y_B^{\text{HET}}}{y_B^{\text{WT}}}\right) = \log_2\left(\frac{(1/2)^{1-C}}{1/2}\right) = C$$

Software

GSNAP 2013-06-27
Samtools 0.1.19
Python 2.7.4
HTSeq 0.5.4p5
R sessionInfo():

```
R version 3.0.2 (2013-09-25)
Platform: x86_64-apple-darwin10.8.0 (64-bit)

locale:
[1] C

attached base packages:
[1] splines   grid      parallel  stats     graphics  grDevices utils
[8] datasets  methods   base

other attached packages:
[1] mgsa_1.10.0           LSD_2.5               ellipse_0.3-8
[4] schoolmath_0.4        colorRamps_2.3        RColorBrewer_1.0-5
[7] MASS_7.3-29           Hmisc_3.13-0          Formula_1.1-1
[10] survival_2.37-4        cluster_1.14.4        gtools_3.2.0
[13] gplots_2.12.1          geneplotter_1.40.0     annotate_1.40.0
[16] AnnotationDbi_1.24.0   lattice_0.20-23        Biobase_2.22.0
[19] genomeIntervals_1.18.0 intervals_0.14.0       epitools_0.5-7
[22] doMC_1.3.2             iterators_1.0.6        foreach_1.4.1
[25] DESeq2_1.2.8           RcppArmadillo_0.4.000  Rcpp_0.10.6
[28] GenomicRanges_1.14.4   XVector_0.2.0          IRanges_1.20.6
[31] BiocGenerics_0.8.0     data.table_1.9.2       rj_1.1.3-1

loaded via a namespace (and not attached):
[1] DBI_0.2-7          KernSmooth_2.23-10 RSQLite_0.11.4     XML_3.95-0.2
[5] bitops_1.0-6       caTools_1.16       codetools_0.2-8    gdata_2.13.2
[9] genefilter_1.44.0  locfit_1.5-9.1     plyr_1.8           reshape2_1.2.2
[13] rj.gd_1.1.3-1      stats4_3.0.2       stringr_0.6.2      tools_3.0.2
[17] xtable_1.7-1
```

Acronyms

ADE allelic differential expression

eQTL expression Quantitative Trait Loci

Supplementary figures

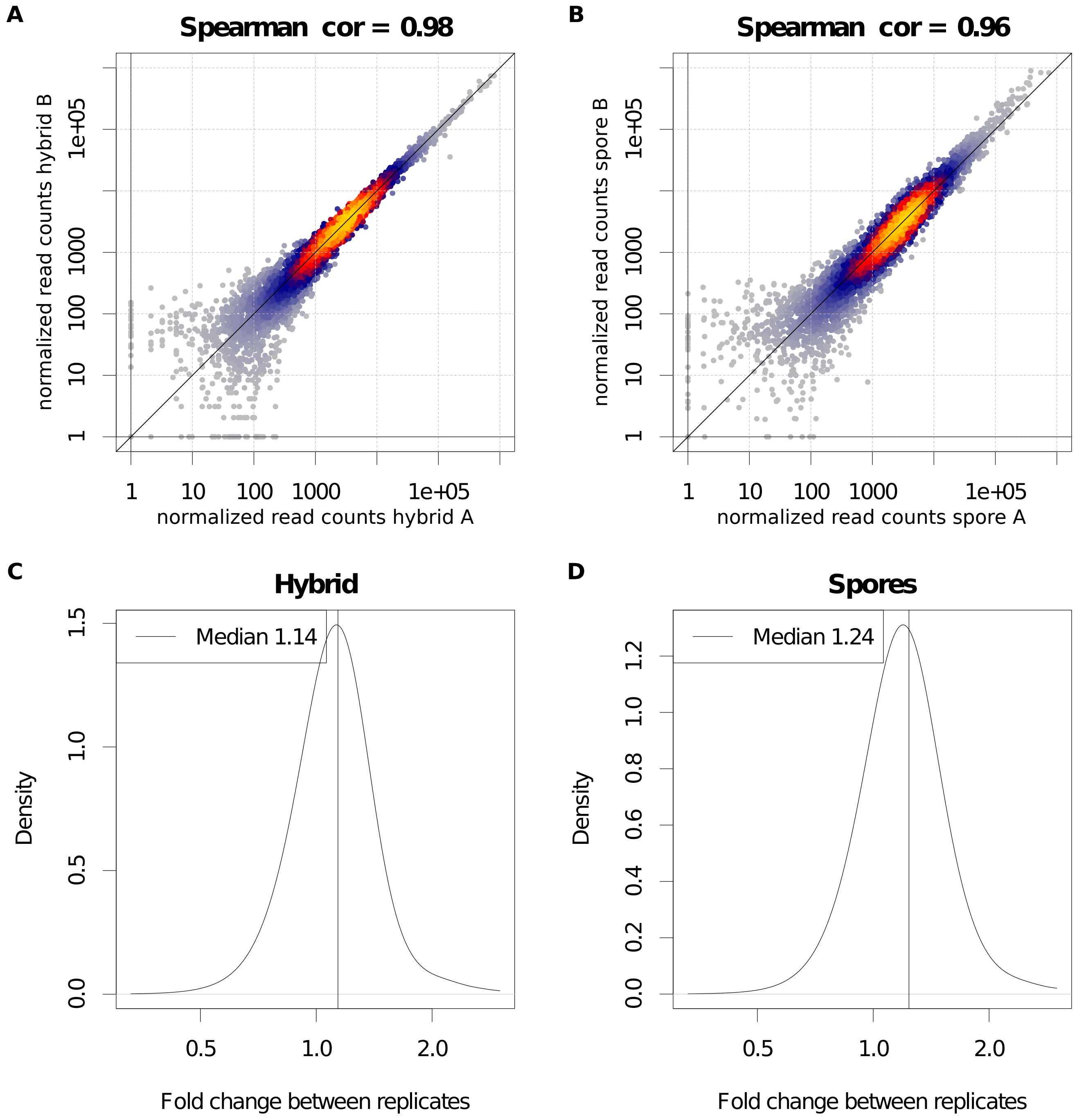

Figure S1: **Biological replicate variation. (A-B)** Scatter plot of gene-level allelic read counts corrected for sequencing depth and genomic allele frequency (Methods) for hybrids (A) and pools of spores (B). **(C-D)** Distribution of the gene-level fold change between the biological replicates for hybrids (C) and pools of spores (D).

Spore pool A

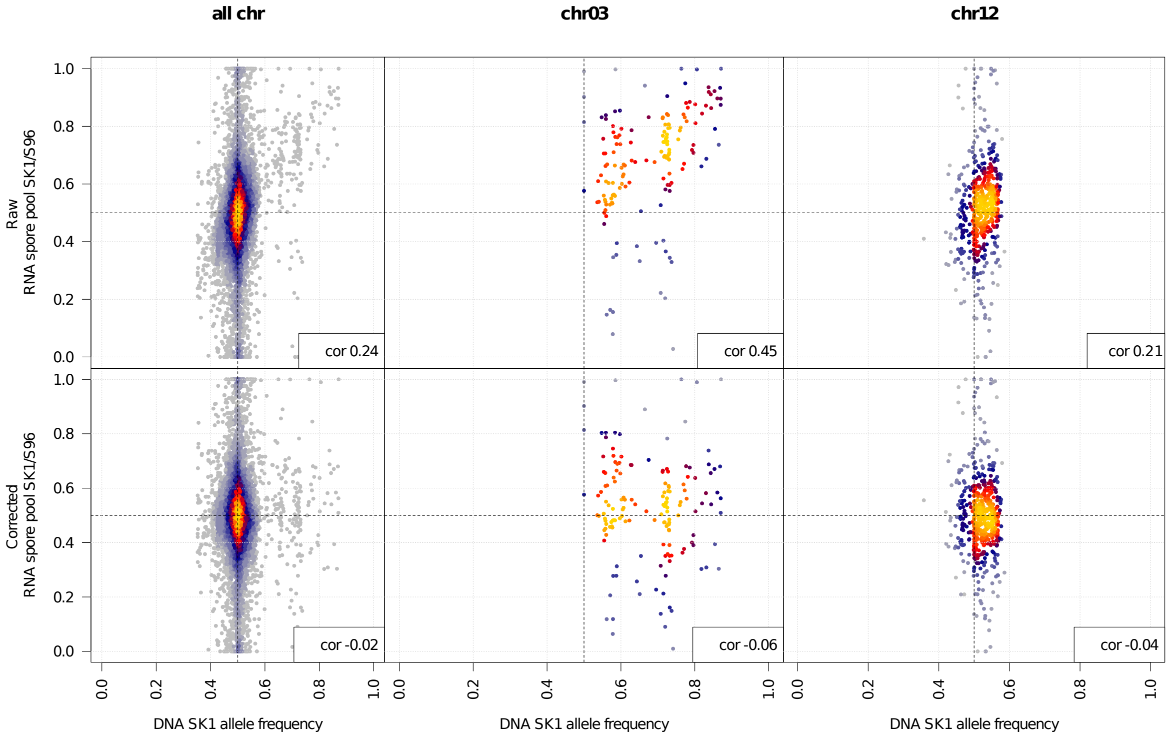

Spore pool B

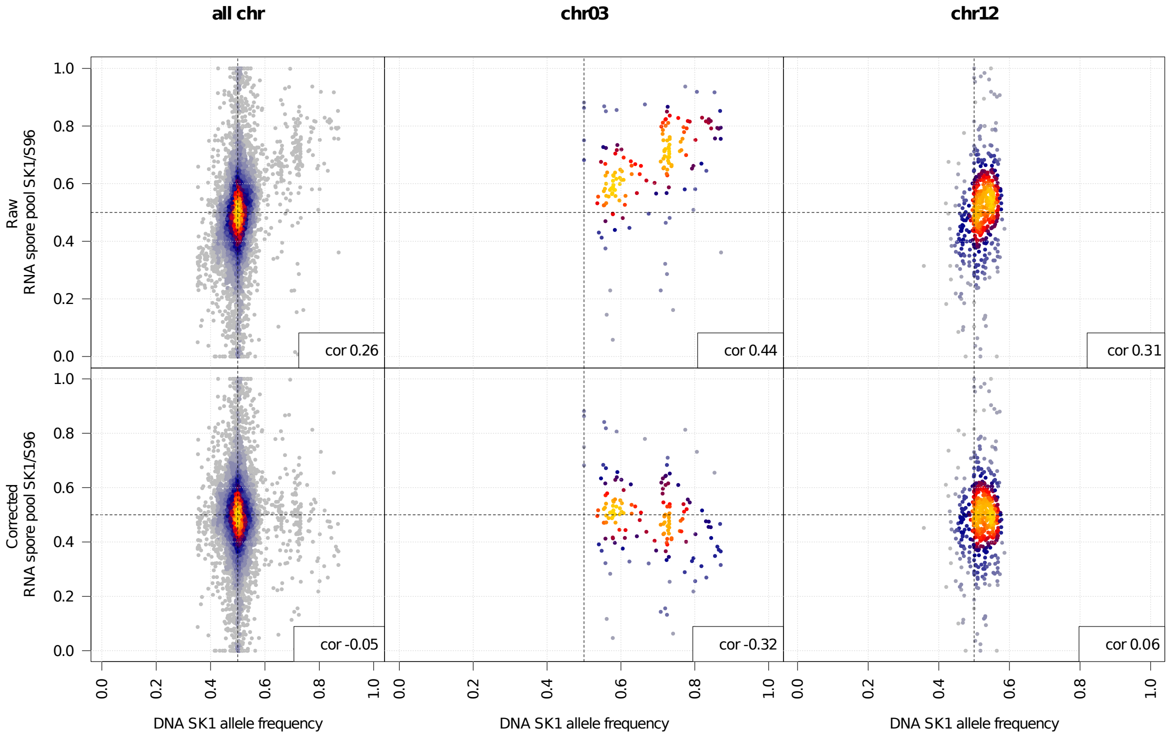

Figure S2: **Correction for genomic allele frequency.** For the spore pool A (top) and for the spore pool B (bottom): RNA count ratios (y-axis, top row) and RNA count ratios corrected for genomic allele frequency (y-axis, bottom row, Methods) versus genomic allele frequency (x-axis) and respective Spearman correlation (lower right corner). Artificial selection (MAT Locus on chromosome III) and natural selection (presumably for $HAP1$ on chromosome XII) leading to genomic allele frequency imbalance in the pool of spores.

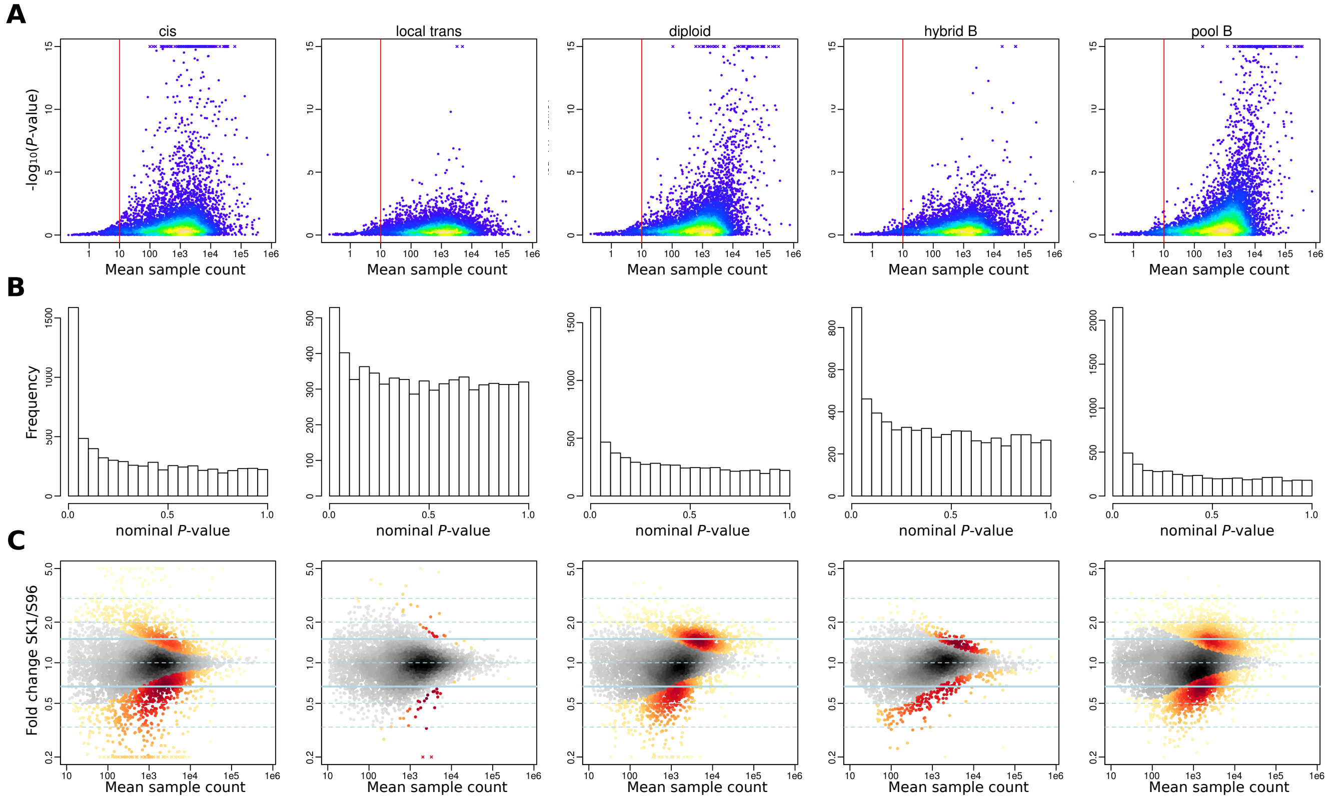

Figure S3: **DESeq2 statistics**. **(A)** Effect of minimum read coverage filter. No small *P*-values (y-axis) at a mean sample count (x-axis) smaller than ten (red vertical line) are reported due to poor statistical power. These genes are filtered out for further analysis. Outlier with $P < 10^{-15}$ are indicated with a cross. **(B)** Histograms of nominal *P*-values. All effects show the expected L-shaped (and not a J-shaped nor U-shaped) distribution of *P*-values indicating that *P*-values are not overestimated. **(C)** Scatter plot of fold change (y-axis) versus mean sample count (x-axis). Genes with an FDR < 0.2 are highlighted (red to yellow). Highlighted genes with a fold change greater than 1.5 (solid light blue line) were considered significant for the corresponding effect (Methods).

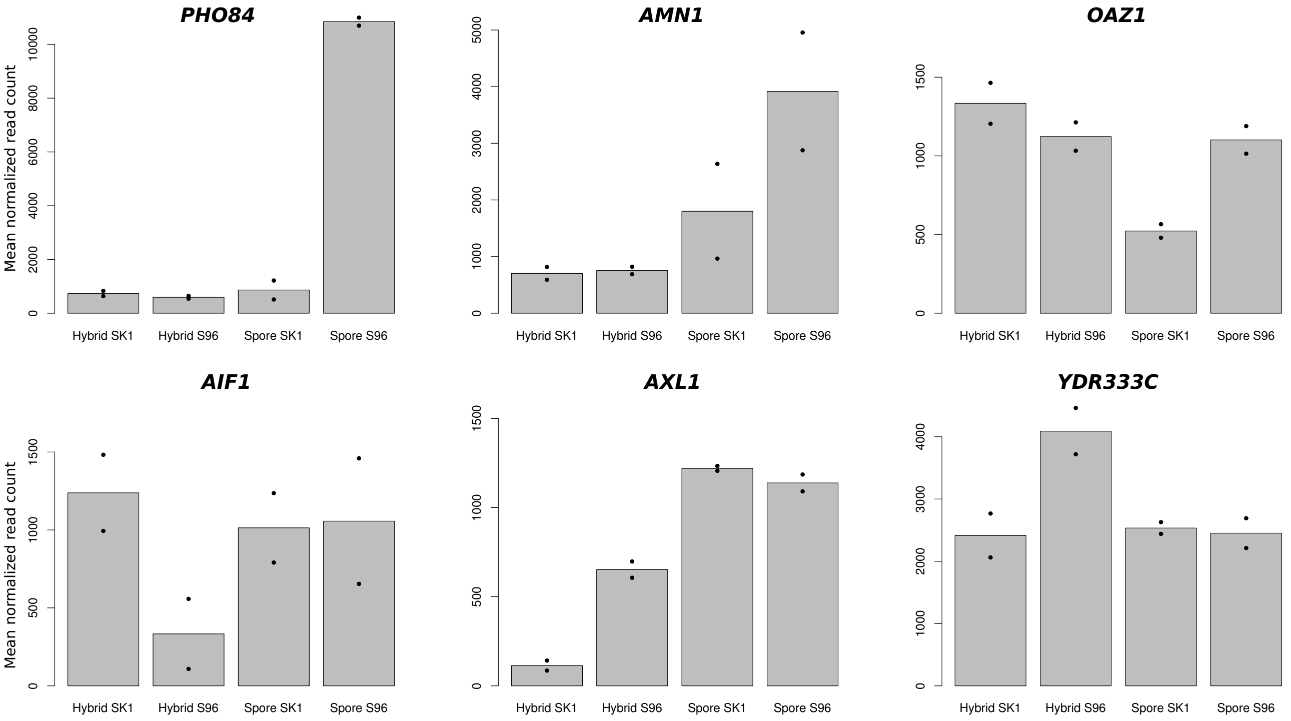

Figure S4: **Read counts corrected for sequencing depth and genomic allele frequency for six local trans genes.** Top row shows three genes with at least 1.5 fold difference between the count ratio (SK1/S96) of the spores, but not for the hybrid. *PHO84* is a reported case of positive feedback (Wykoff *et al*, 2007; Gagneur *et al*, 2009), which leads to ADE in the pool of spores but not in the hybrid. *AMN1* is known to regulate itself through a negative feedback loop and to carry a coding mutation in the reference lab strain that impairs this feedback (Ronald *et al*, 2005). In the case of a mutation affecting the negative feedback lopp itself, negative feedback is exerted only in the half of the spore population that inherited the functional feedback. Thus allelic differential expression is specific or at least stronger in the pool of spores than in the hybrid. Consistently, *AMN1* showed only allelic differential expression in the spores. Bottom row shows three genes with at least 1.5 fold difference between the count ratio of the hybrid strains, but not for the spores. Individual replicate measures are indicated by black dots.

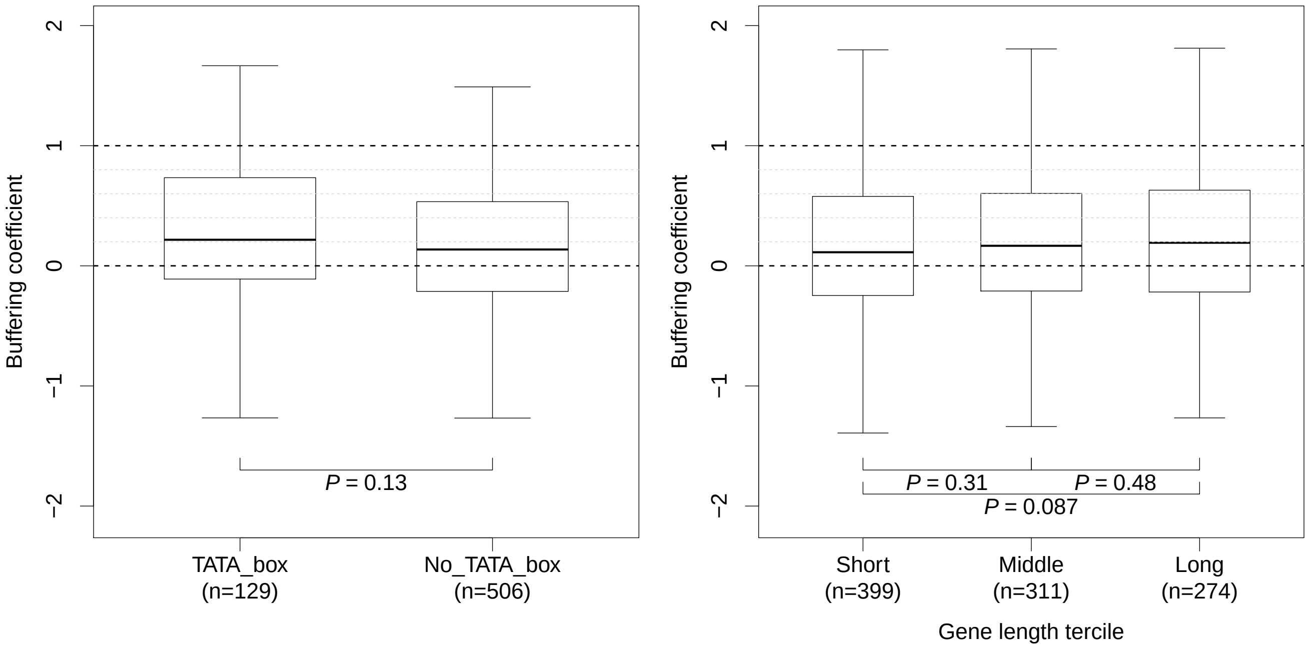

Figure S5: **Buffering compared to gene features**. No correlation between buffering coefficient and TATA box presence as well as gene length (Lundberg *et al*, 2012) defined as mean of SK1 and S96 length among cis genes. *P*-values were computed using a two-sided Wilcoxon test.

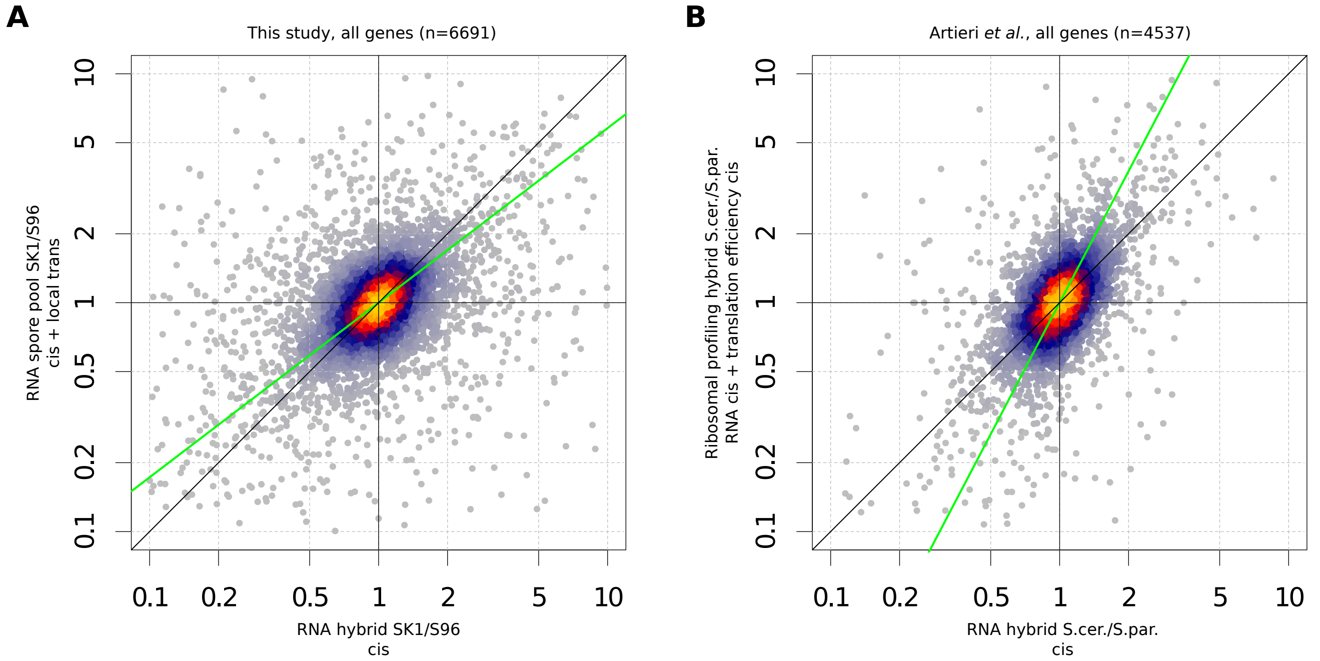

Figure S6: **Analysis of buffering trend across all genes (A)** Scatterplot of allelic ratio corrected for sequencing depth and genomic allele frequency in pool of spores (y-axis) against the hybrid (x-axis) for all genes. We used principal component analysis to estimate buffering across all genes because the buffering coefficient is ill-defined for non-cis genes (Methods). The trendline (green) is the direction of the first principal component. Its slope (0.75) is lower than 1 indicating genome-wide trend for buffering of cis effects by local trans effects. Note that this analysis is conservative since larger replicate variance for the pool of spores (y-axis) than for the hybrid (x-axis) leads to overestimation of the first pincipal component slope. **(B)** Scatterplot of allelic ratio in ribosome profiling data (y-axis) against allelic ratio in ribosome profiling data (x-axis) in the *S. cerevisiae* x *S. paradoxus* hybrid for all genes (data from Artieri and Fraser (2014)). The first principal component is above diagonal (slope=1.90, green line), thus does not provide evidence for buffering genome-wide at the translational level. Here, the slope overestimation of the principal component analysis might confound this result. Moreover, because variance between biological replicates is larger across RNAseq for pools of spores than for ribosomal profiling across hybrids, a buffering effect as large as the one seen in the spores would have been detected, if it were present at the translational level.

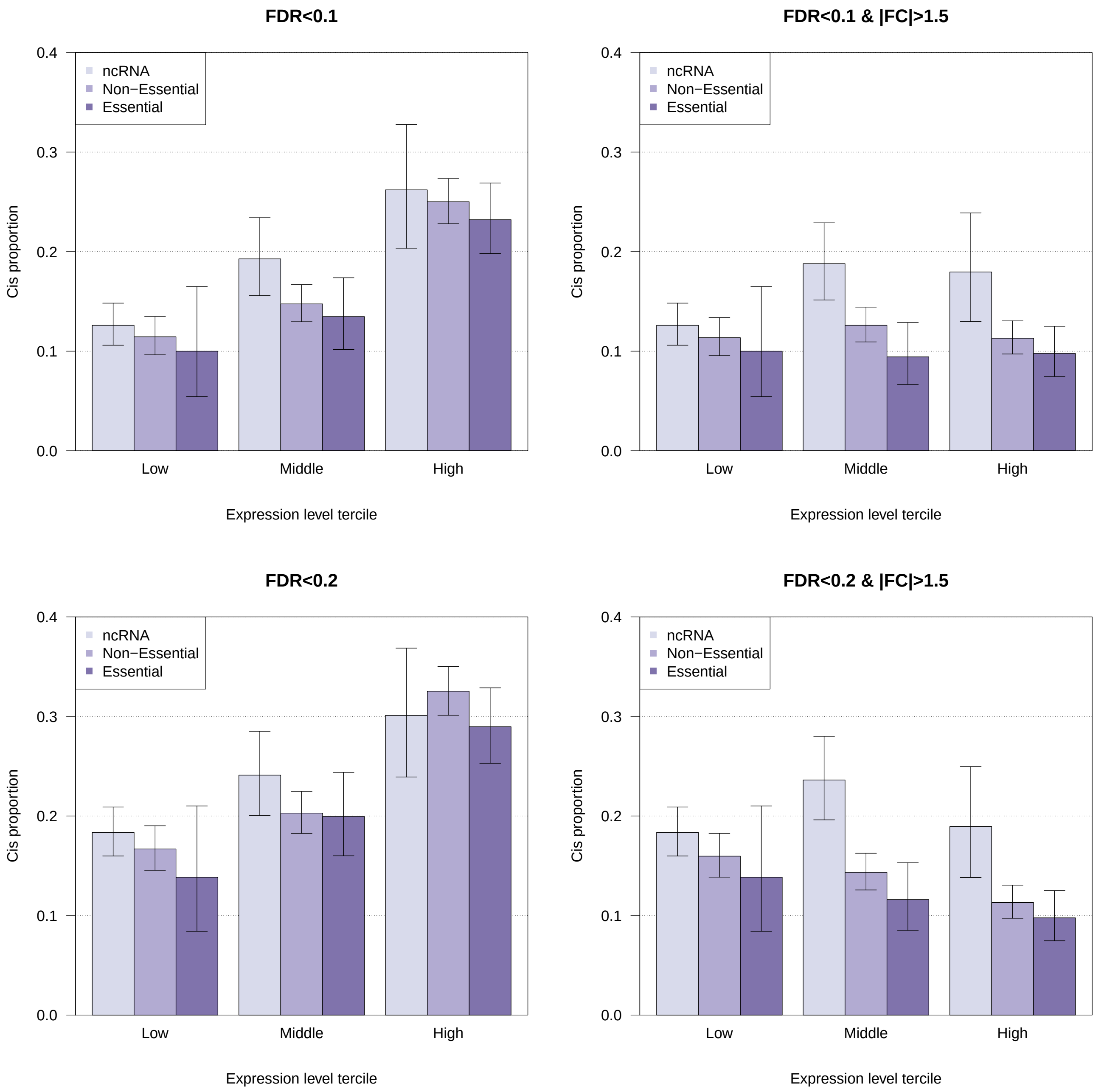

Figure S7: **Correlation between cis detection and expression.** Analog to Fig 3A: proportion of cis genes for gene categories (purple shadings) and expression level terciles (grouped bars). The applied thresholds for false discovery rate (FDR) and absolute fold change (|FC|) are indicated for each plot (title).

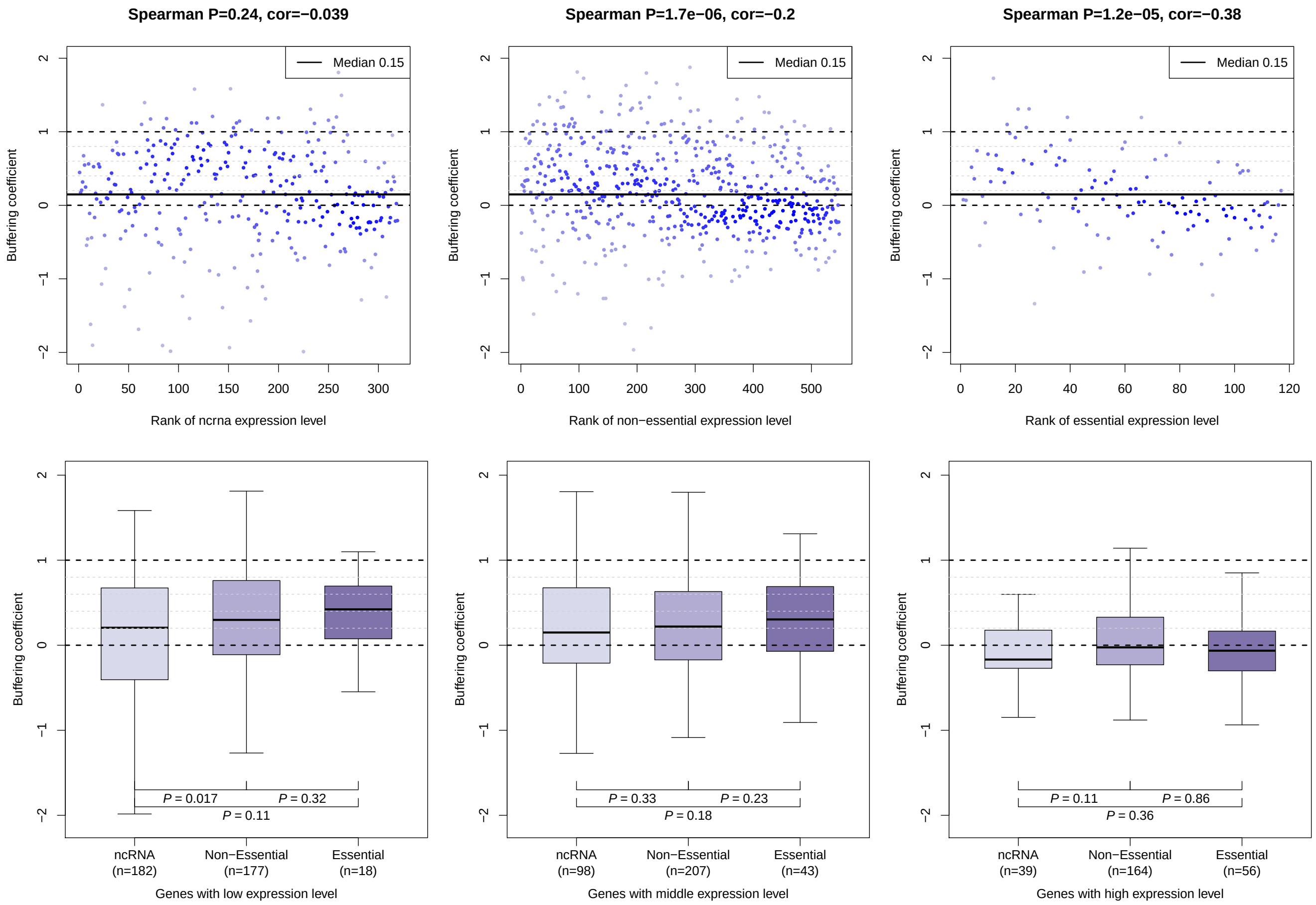

Figure S8: **Detailed figure 3C and 3D**. Buffering coefficient compared against the ranks of expression level for all gene categories (top). A significant one-sided Spearman correlation test (caption) confirmed the trend in Fig 3C as well as for Fig 3D (one-sided Wilcoxon test, bottom)

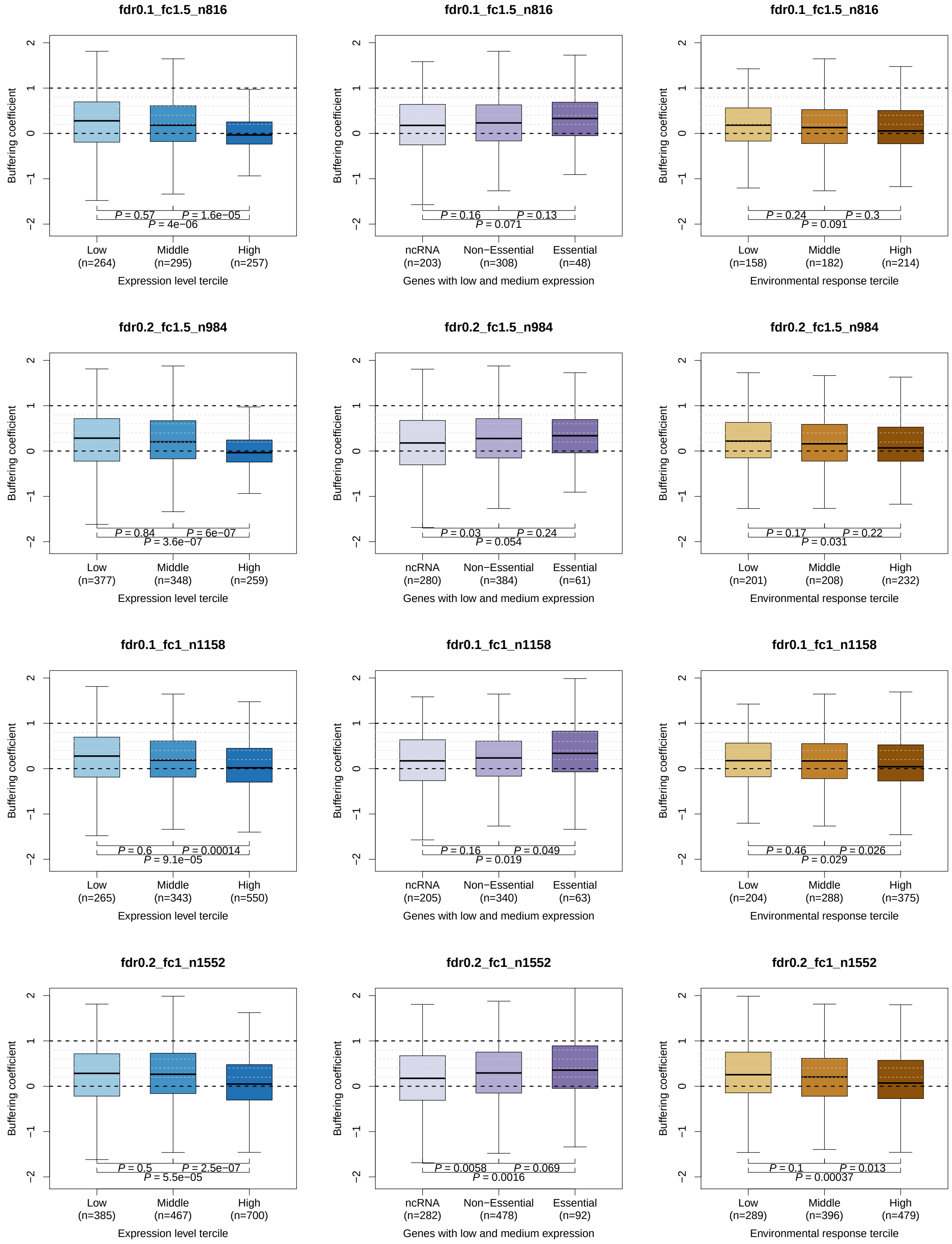

Figure S9: **Evaluation of different thresholds.** The figures 3C, 3D and 4C (left to right) are generated for different sets of cis genes. These cis gene sets vary in the false discovery rate (fdr) and absolute fold change (fc) filter that was applied (title) and therefore also in size (n, increasing top to bottom).

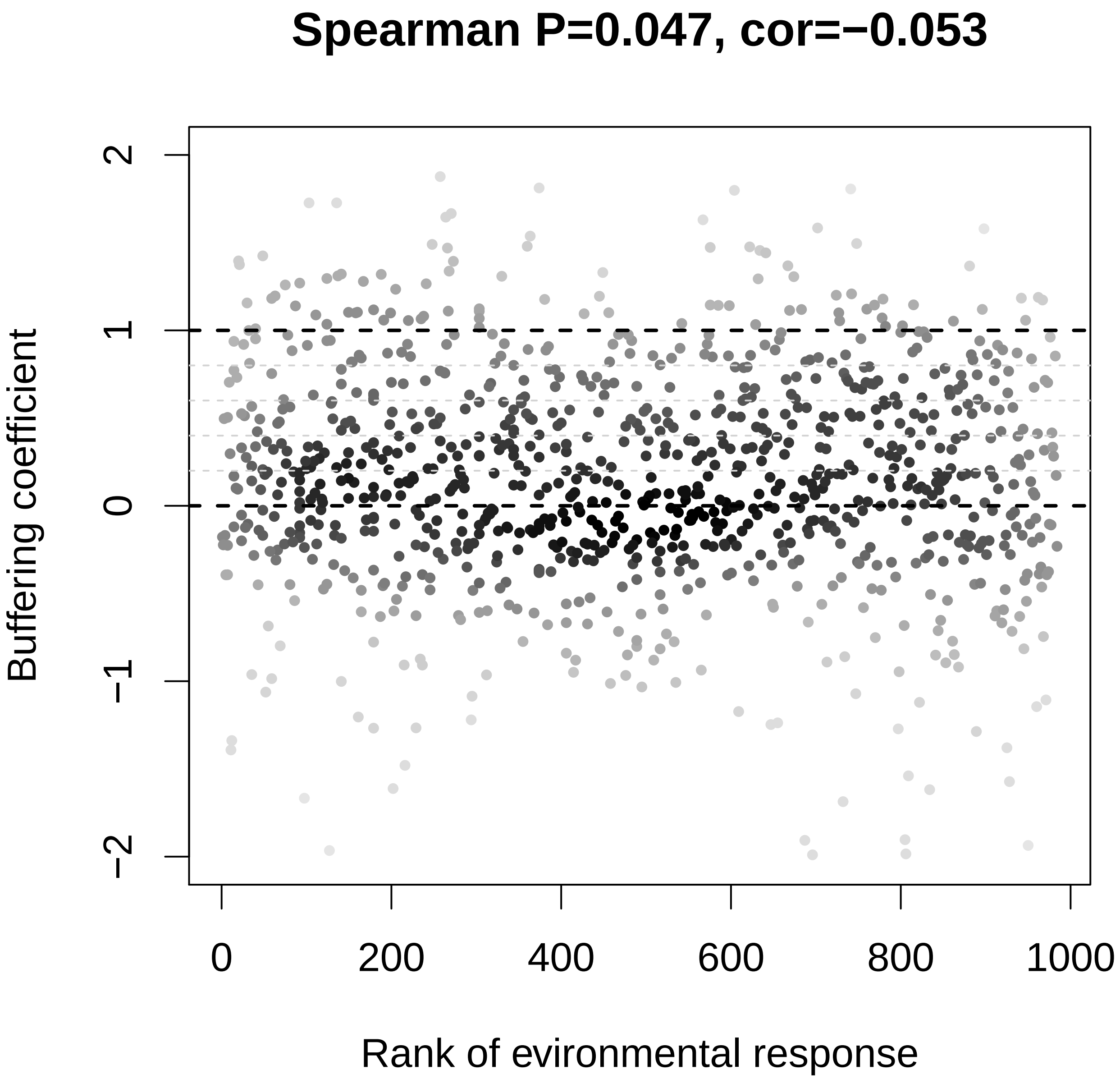

Figure S10: **Detailed figure 4C.** Buffering coefficient compared against the ranks of environmental response (Tirosh *et al*, 2009). A one-sided Spearman correlation test (title) confirmed the trend in Fig 4C.

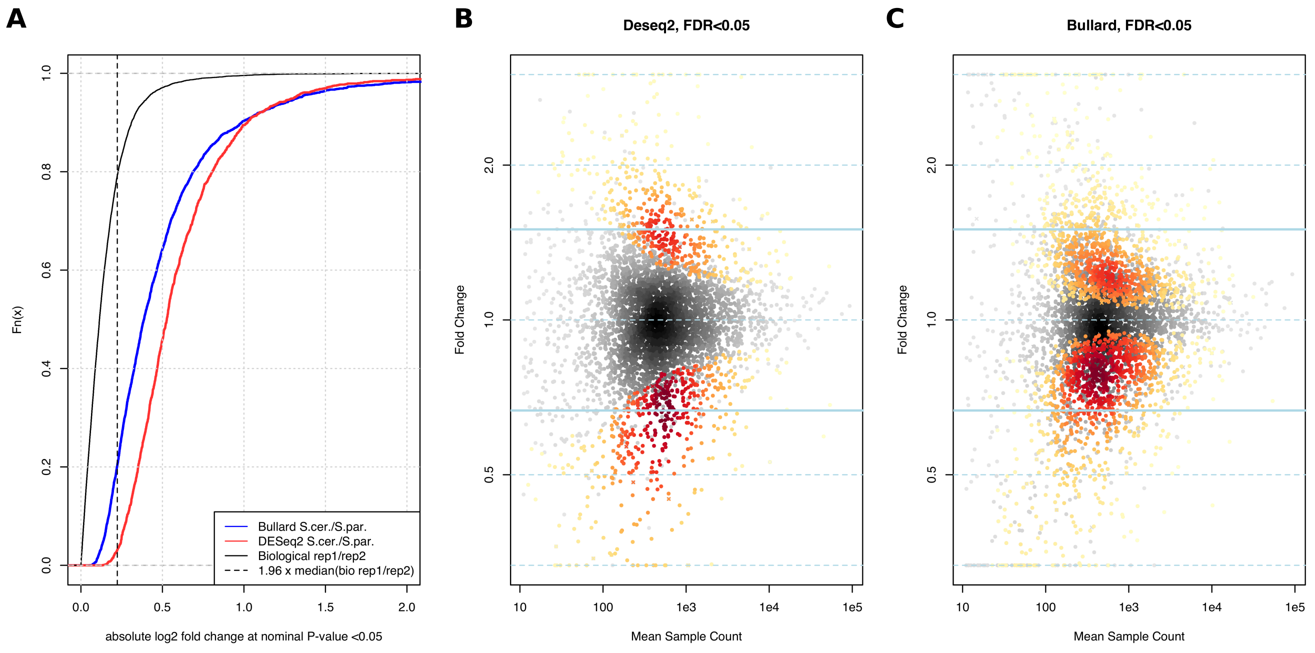

Figure S11: **Comparison of DESeq2-based test for ADE and the test described by Bullard *et al* (2010). (A)** Empirical cumulative distribution of $\log_2$-fold change of RNA expression level between the two biological replicates in the *S. cer* x *S. par* hybrid (black), 1.96 times the median standard deviation of $\log_2$-expression levels across biological replicates in the same hybrid (vertical dashed line), $\log_2$-fold change of allelic expression ratio among genes with a significant allelic differential expression at a nominal *P*-value of 0.05 according to the originally used statistical test based on Bullard *et al* (2010) (blue) and according to the approach developed here based on DeSeq2 (red). **(B)** Same as Supplementary Fig S3C, but the FDR threshold of 0.05 (red) is used instead of 0.2 to match the cutoff used by Artieri and Fraser (2014) based on Bullard test. **(C)** Same as (B) using the originally used statistical test based on Bullard *et al* (2010).

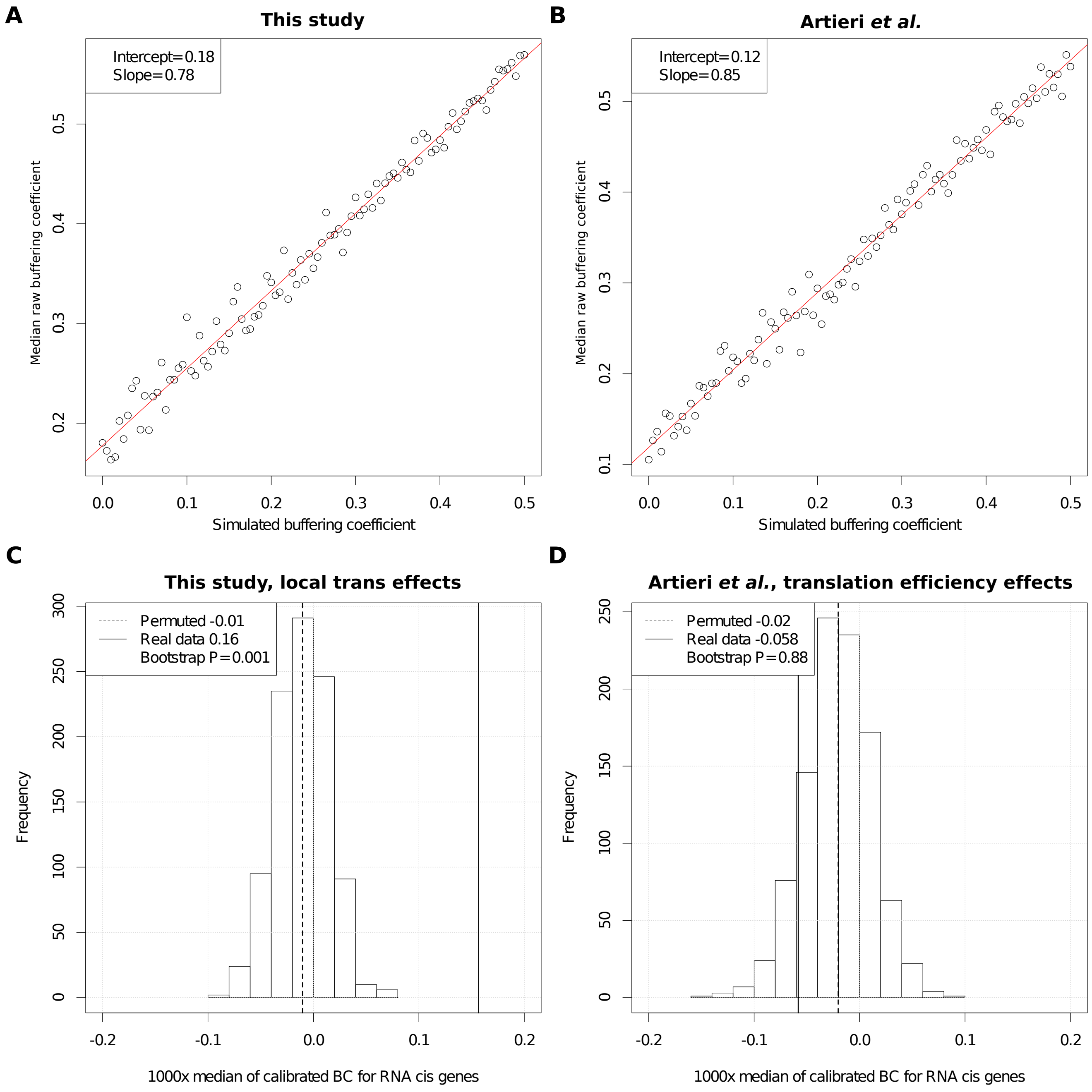

Figure S12: **Calibration of buffering coefficient and testing.** **(A)** Simulated genome-wide buffering coefficient (x-axis, Methods) versus median observed raw buffering coefficient (y-axis). Linear regression (red line) is used to calibrate observed raw buffering coefficients. **(B)** Analogous to (A), but for Artieri and Fraser (2014) data (Methods). Here too, a linear model gives a good calibration. **(C)** Distribution of median buffering coefficient across genes under independence of cis and local trans effects (1,000 permutations and dataset simulations, methods). The distribution is centered at zero (dashed line, median=-0.01) confirming with a distinct simulation scheme the correctness of the calibration. The observed median buffering coefficient (solid black line) is larger than on any dataset simulated under independence assumption (Bootstrap *P*-value = 0.001). **(D)** Analogous to (C), but for Artieri and Fraser (2014) data (Methods). The distribution is centered at zero (dashed line, median=-0.02) confirming with a distinct simulation scheme the correctness of the calibration. However, the observed median buffering coefficient (solid black line) is not significantly large (Bootstrap *P*-value = 0.88).

References

Albert FW, Muzzey D, Weissman JS, Kruglyak L (2014) Genetic Influences on Translation in Yeast. *PLoS genetics* **10**: e1004692

Artieri CCG, Fraser HBH (2014) Evolution at two levels of gene expression in yeast. *Genome research* **24**: 411–21

Bullard JH, Mostovoy Y, Dudoit S, Brem RB (2010) Polygenic and directional regulatory evolution across pathways in Saccharomyces. *Proceedings of the National Academy of Sciences of the United States of America* **107**: 5058–63

Gagneur J, Sinha H, Perocchi F, Bourgon R, Huber W, Steinmetz LM (2009) Genome-wide allele- and strand-specific expression profiling. *Molecular Systems Biology* **5**: 274

Lundberg LE, Figueiredo MLa, Stenberg P, Larsson J (2012) Buffering and proteolysis are induced by segmental monosomy in Drosophila melanogaster. *Nucleic acids research* **40**: 5926–37

McManus CJ, Coolon JD, Duff MO, Eipper-Mains J, Graveley BR, Wittkopp PJ (2010) Regulatory divergence in Drosophila revealed by mRNA-seq. *Genome research* **20**: 816–25

McManus CJ, May GE, Spealman P, Shteyman A, McManus J (2014) Ribosome profiling reveals post-transcriptional buffering of divergent gene expression in yeast. *Genome research* **24**: 422–30

Ronald J, Brem RB, Whittle J, Kruglyak L (2005) Local regulatory variation in Saccharomyces cerevisiae. *PLoS genetics* **1**: e25

Springer M, Weissman JS, Kirschner MW (2010) A general lack of compensation for gene dosage in yeast. *Molecular Systems Biology* **6**: 368

Tirosh I, Reikhav S, Levy Aa, Barkai N (2009) A yeast hybrid provides insight into the evolution of gene expression regulation. *Science New York NY* **324**: 659–62

Wykoff DD, Rizvi AH, Raser JM, Margolin B, O'Shea EK (2007) Positive feedback regulates switching of phosphate transporters in S. cerevisiae. *Molecular cell* **27**: 1005–13
