# Supplementary material for: Negative feedback buffers effects of regulatory variants
Source: Mol Syst Biol. 2015 Jan 29;11(1):785. doi: 10.15252/msb.20145844 (PMC4332157; doi:10.15252/msb.20145844)
Supplement: Supplementary file 5 [file msb0011-0785-sd5.pdf]

Negative feedback buffers effects of regulatory variants

Daniel M. Bader, Stefan Wilkening, Gen Lin, Manu Tekkedil, Kim Dietrich, Lars M. Steinmetz and Julien Gagneur

Corresponding author: Julien Gagneur, Ludwig-Maximilians University Munich

Review timeline:

Submission date:	15 October 2014
Editorial Decision:	26 November 2014
Revision received:	19 December 2014
Accepted:	23 December 2014

Editor: Thomas Lemberger

Transaction Report:

(Note: With the exception of the correction of typographical or spelling errors that could be a source of ambiguity, letters and reports are not edited. The original formatting of letters and referee reports may not be reflected in this compilation.)

1st Editorial Decision 26 November 2014

Thank you again for submitting your work to Molecular Systems Biology. We have now heard back from the three referees who accepted to evaluate the study. As you will see, the referees find the topic of your study of potential interest and are supportive. They raise however a series of concerns and make suggestions for modifications, which we would ask you to carefully address in a revision of the present work. The recommendations provided by the reviewers are very clear in this regard and refer mainly to additional clarifications and the need to word some of the conclusions more carefully.

Reviewer #1:

The authors use an elegant and efficient experimental design using RNA-seq in a hybrid diploid yeast and pools of halpoid segregants to distinguish between genetic variants close ('local') to a gene that influence expression in cis and trans. This allows them to reach the conclusion that many genes regulate there own expression by feedback ('tho the mechanisms are obscure). They also re-analyse ribosome profiling data that was previously used to suggest buffering of changes in RNA expression via changes in translation. They find no evidence for this but rather show the previous result is likely due to a technical artefact in the data processing.

Overall I found this is a very interesting study that systematically addresses a basic and important question in gene regulation genome-wide.

Minor suggestions:

In the analysis with respect to gene importance the authors (need to) control for expression level. It would be re-assuring to see this elsewhere. e.g. is there a bias in the detection of local-trans or local-

cis eQTLs depending upon expression levels in the pools and does this effect the results?

Molecular mechanisms - (for good reason) the authors speculate little about possible molecular mechanisms. But it would be interesting to know a bit more about the properties/functions of the genes they do detect feedback for - what functional categories/age of proteins do they cover etc?

Fig 1B - I don't understand the choice of example shown here. If I understand correctly in this example the example is not any of their classes in fig 1C (looks like the expression levels are swapped between the diploids and haploids?)

Abstract - delete the word 'major' in the last sentence. Similarly in the first sentence of the intro and 'vast' in the second!

Reviewer #2:

Bader et al. compare allele-differential expression (ADE) in hybrid (diploid) yeast and in pools of their (haploid) progeny to distinguish three possible causes of ADE: 1) cis-regulatory variation, 2) local trans-factor variation, and 3) distant trans-factor variation. Local trans variation includes negative feedback of a gene's product on its own expression. The authors conclude that local trans variation is substantial and likely reflects negative feedback. They also conclude, contrary to two prior studies, that there is little evidence for buffering of gene-expression differences by compensatory changes in translation efficiency. This a clever, rigorously performed, well presented and convincing study that is certainly appropriate for publication in Molecular Systems Biology.

Mechanisms that buffer the effects of environmental or genetic differences on phenotypes, including gene-expression phenotypes, are of broad interest in a range of fields, from developmental biology to evolutionary biology to systems biology to human genetics. ADE is a way to look at how cis-regulatory variation that affects transcript levels may be buffered. As noted above, prior studies compared ADE and allele-differential translation efficiency, and concluded that ADE is buffered at the translational level. Bader et al.'s novel experimental design allowed a test of whether ADE is buffered by negative feedback at the transcriptional level. Specifically they can distinguish trans regulatory variation that is local to the gene of interest (either in the gene itself or in a neighboring gene) from trans regulatory variation that is distant from the gene of interest. They find a substantial number of cases of local trans variation and find, using data from another prior study that measured expression in homozygous wild-type vs. heterozygous yeast, that the likely mechanism is negative feedback. The logic behind these analyses is sound and clearly presented, and the data support the conclusions. In particular, care was taken to avoid various pitfalls in analyzing the RNA-seq data. The authors also convincingly critique the prior studies that claimed compensation at the level of translation efficiency.

The following suggestions are not meant to take away from the strength of the manuscript but are offered as ways to improve it:

1) Although the manuscript is generally well written, there are some lapses in precision. For example, on page 4: "genes localizing in the extracellular region" and "genes were not transcription factors" should be, respectively, "genes encoding proteins that localize in the extracellular region" and "genes that do not encode transcription factors". Also, on page 10: "The false discovery rate ... for each of the resulting 6,681 genes" is not technically correct. The FDR is a property of a set of genes called significant, not of a single gene. So the statement should instead be something like "The minimum false discovery rate at which each of the resulting 6,681 genes would be called significant".

2) The analysis uses some arbitrary thresholds, such as (page 4) > 1.5-fold differences in expression between alleles and FDR < 0.2. It would be good to report variations on the analysis using different thresholds to show that the analysis is robust to these arbitrary decisions.

3) Several of the analyses classify genes into low, middle and high expression terciles, and statistical tests are performed to test for differences between these terciles. It would be good to see the data presented in all their quantitative detail rather than just binned like this. For example, scatterplots of

the data that went into Figures 3C and 4A would be useful. Similarly, Figure 3D aims to show a connection between the inferred fitness relevance of a gene, the gene's expression level and the buffering coefficient. A scatterplot could also be used here, and the statistical analysis performed using a method that considers the three dimensions (and their correlations) simultaneously.

4) In the Discussion (page 7) the authors state that "Hsp90 confers mutational robustness". This is a common mistake, but no experiment has yet been reported that tested whether Hsp90 buffers the effects of new mutations. Hsp90 impairment can reveal cryptic genetic variation, but this is not the same thing as lowering mutational robustness. Moreoever, in some cases Hsp90 impairment suppresses variation (leading to the inference that Hsp90 is a potentiator of variation rather than a capacitor). For an explanation of why cryptic genetic variation and mutational buffering are not necessarily related, see Hermisson & Wagner (2004). Also, for an argument against chaperones buffering mutations "by facilitating the correct folding of proteins" (as stated by the authors), see Bobula et al. (2006).

5) The Masel & Siegal (2009) review cited by the authors presents an argument about negative feedback and buffering environmental fluctuations vs. genetic variation that seems relevant to this manuscript. In particular, the review argues that negative feedback more generally buffers environmental fluctuations, but does not necessarily buffer genetic variation. But if the negative feedback operates through a highly cooperative mechanism then buffering of genetic variation would be more likely. Because the local trans variation seems not to be affecting transcription factor encoding genes, it might be difficult to test this idea about cooperativity but maybe the authors can think of something?

Reviewer #3:

Bader and colleagues have designed an elegant experimental systems to detect RNA expression buffering in hybrid strains. This systems compares allele expression in hybrid strains to those in a pool of spores. Using this approach the authors report their failure to detect signs of translation buffering described earlier and point at negative feedback as an important mechanism of allele buffering. Coming from an experimental background, I must confess that I had hard time getting my head around this paper. The experimental design is certainly elegant. However, the lack of follow up experiment makes it difficult for me to unequivocally endorse the authors' claims. My main problem is with "negative feedback" being pushed as a major mechanism of allele buffering. It is really not clear to me, for instance, how one can differentiate between negative feedback and another type of mechanism from the data alone in absence of functional follow up. This may be down to me not being an expert in the field, but many MSB readers won't be either. At the very least, the authors need to make it clearer why they believe their conclusions on the importance of negative feedback valid in absence of any functional confirmation. Second, the authors reanalyse a ribosome profiling dataset and dismiss previous conclusions that adjustment of translation efficiency is playing an important role in allele buffering. Here Bader and colleague have the burden of proof. What are the evidence that their computational approaches are sensitive enough to detect the effects publishes previously? Ribosomal profiling data are quite different from RNA-seq data, regarding transcript coverage for instance, how does it impact on the sensitivity of their statistical analysis? From looking at figure 2C quite a few genes must have a $C > 0$, why are these not relevant?
I also have a couple of specific points that require clarification:
- Average allele specific reads per sample is around 10. I suppose that median number is lower. It is not clear to me how these relatively low numbers will affect conclusions in figure 3 where a relationship between transcripts expression levels and ADE is observed. What are the evidence that their approach in not biased by the number of diagnostic reads for each allele? This is also relevant for the ribosomal foot-printing data where numbers of allele specific reads are likely to be low.

- "Together, these findings indicate that buffering through local trans regulation had been underestimated so far.". It is not clear to me where previous estimates were published.

1st Revision - authors' response 19 December 2014

Molecular Systems Biology – Revision MSB-14-5844

Negative feedback buffers effects of regulatory variants

Point by point response

Reviewer 1

The authors use an elegant and efficient experimental design using RNA-seq in a hybrid diploid yeast and pools of halpoid segregants to distinguish between genetic variants close ('local') to a gene that influence expression in cis and trans. This allows them to reach the conclusion that many genes regulate there own expression by feedback ('tho the mechanisms are obscure). They also re-analyse ribosome profiling data that was previously used to suggest buffering of changes in RNA expression via changes in translation. They find no evidence for this but rather show the previous result is likely due to a technical artefact in the data processing.

Overall I found this is a very interesting study that systematically addresses a basic and important question in gene regulation genome-wide.

R1.1

In the analysis with respect to gene importance the authors (need to) control for expression level. It would be re-assuring to see this elsewhere. e.g. is there a bias in the detection of local-trans or local-cis eQTLs depending upon expression levels in the pools and does this effect the results?

A: Indeed, detection of cis and local trans effects depends on the level of expression. Inherently to the sequencing assay, detection of local-trans and cis effects by pure statistical significance is more sensitive in the high range of expression. Cis effects can be detected even at low fold-changes for the highly expressed genes but not for the lowly expressed genes (Supplementary Fig S3C). This will result in a bias for coding, essential genes. This bias is our motivation for combining a fold-change cutoff with a *P*-value cutoff. We agree with reviewer 1 that it is important to show that our results do not depend on expression level and on this cutoff combination. We now control for expression levels when assessing the proportion of cis effects by gene category which confirmed the genome-wide trend (Supplementary Fig S7). There are too few local trans genes to perform further stratification (54 genes). Nonetheless, we checked the distribution of expression among local trans genes across the terciles (low 10, middle 22, high 22 genes) which is slightly, but not significantly enriched for middle to high expression levels (Multinomial test, *P*=0.06). Moreover, in response to a related request from reviewer 2, we demonstrate now that our conclusions on buffering are not affected by levels of expression, nor by the choice of FDR (0.1 or 0.2) or the application of a fold change cutoff (Supplementary Fig S9).

R1.2

Molecular mechanisms - (for good reason) the authors speculate little about possible molecular mechanisms. But it would be interesting to know a bit more about the properties/functions of the genes they do detect feedback for - what functional categories/age of proteins do they cover etc?

A: We performed gene ontology enrichment analysis for the buffered genes (cis genes with buffering coefficient greater zero) but lacked enriched terms. This result is now reported (2nd result section, 1st paragraph). We also lacked significant association wit gene features that have been associated with gene expression variability (TATA box) and dosage compensation in fly (gene length) (2nd result section, end of first paragraph and Supplementary Fig S5). Together with the fact that local trans buffering is stronger for essential genes, this indicates that local trans buffering is general mechanism found across many pathways and distinct from aforementioned mechanisms.

R1.3

Fig 1B - I don't understand the choice of example shown here. If I understand correctly in this example the example is not any of their classes in fig 1C (looks like the expression levels are swapped between the diploids and haploids?)

A: The former sketch was indeed misleading. We modified figure 1B to depict a partial buffering scenario and now explicitly provide the interpretation of figure 1B in the introduction (4th paragraph).

R1.4

Abstract - delete the word 'major' in the last sentence. Similarly in the first sentence of the intro and 'vast' in the second!

A: We corrected abstract and introduction as suggested and exchanged the word “major” in the last sentence of the introduction with “important”. However, we kept the first sentence of the introduction, since it describes regulatory variants per se and not buffering (“Regulatory genetic variants play a major role in phenotypic variation and evolution.”).

Reviewer 2

Bader et al. compare allele-differential expression (ADE) in hybrid (diploid) yeast and in pools of their (haploid) progeny to distinguish three possible causes of ADE: 1) cis-regulatory variation, 2) local trans-factor variation, and 3) distant trans-factor variation. Local trans variation includes negative feedback of a gene's product on its own expression. The authors conclude that local trans variation is substantial and likely reflects negative feedback. They also conclude, contrary to two prior studies, that there is little evidence for buffering of gene-expression differences by compensatory changes in translation efficiency.

This a clever, rigorously performed, well presented and convincing study that is certainly appropriate for publication in Molecular Systems Biology.

Mechanisms that buffer the effects of environmental or genetic differences on phenotypes, including gene-expression phenotypes, are of broad interest in a range of fields, from developmental biology to evolutionary biology to systems biology to human genetics. ADE is a way to look at how cis-regulatory variation that affects transcript levels may be buffered. As noted above, prior studies compared ADE and allele-differential translation efficiency, and concluded that ADE is buffered at the translational level. Bader et al.'s novel experimental design allowed a test of whether ADE is buffered by negative feedback at the transcriptional level. Specifically they can distinguish trans regulatory variation that is local to the gene of interest (either in the gene itself or in a neighboring gene) from trans regulatory variation that is distant from the gene of interest. They find a substantial number of cases of local trans variation and find, using data from another prior study that measured expression in homozygous wild-type vs. heterozygous yeast, that the likely mechanism is negative feedback. The logic behind these analyses is sound and clearly presented, and the data support the conclusions. In particular, care was taken to avoid various pitfalls in analyzing the RNA-seq data. The authors also convincingly critique the prior studies that claimed compensation at the level of translation efficiency.

The following suggestions are not meant to take away from the strength of the manuscript but are offered as ways to improve it:

R2.1

Although the manuscript is generally well written, there are some lapses in precision. For example, on page 4: "genes localizing in the extracellular region" and "genes were not transcription factors" should be, respectively, "genes encoding proteins that localize in the extracellular region" and "genes that do not encode transcription factors". Also, on page 10: "The false discovery rate ... for each of the resulting 6,681 genes" is not technically correct. The FDR is a property of a set of genes called significant, not of a single gene. So the statement should instead be something like "The minimum false discovery rate at which each of the resulting 6,681 genes would be called significant".

A: We adapted the first two sentences as suggested and modified the passage regarding the multiple testing procedure (Methods “Statistical Modeling”, last paragraph).

R2.2

The analysis uses some arbitrary thresholds, such as (page 4) > 1.5-fold differences in expression between alleles and FDR < 0.2. It would be good to report variations on the analysis using different thresholds to show that the analysis is robust to these arbitrary decisions.

A: We added two figures (Supplementary Fig S7, S9) addressing this issue. We now considered the combination of two possible FDR thresholds (0.2 as originally and a more stringent value 0.1), together with fold change cutoff 1.5 and without. The fold change affects the class of genes with cis effects (Supplementary Fig S3C), due to a

strong bias in detecting power for highly expressed genes when no fold-change cutoff is applied (see also response to R1.1). Yet, our conclusions are not affected by the choice of thresholds.

R2.3

Several of the analyses classify genes into low, middle and high expression terciles, and statistical tests are performed to test for differences between these terciles. It would be good to see the data presented in all their quantitative detail rather than just binned like this. For example, scatterplots of the data that went into Figures 3C and 4A would be useful. Similarly, Figure 3D aims to show a connection between the inferred fitness relevance of a gene, the gene's expression level and the buffering coefficient. A scatterplot could also be used here, and the statistical analysis performed using a method that considers the three dimensions (and their correlations) simultaneously.

A: Figure 3D is now presented in more detail (Supplementary Fig S8 bottom). Moreover, we now provide scatterplots between expression level and buffering for three functional categories separately (Supplementary Fig S8 top). For each category buffering negatively correlates with expression level. Similarly, significant negative correlation is found between buffering and environmental response (Supplementary Fig S10). Complementary, we looked into the distribution of buffering coefficient per gene category stratified by expression level tercile confirming that buffering positively correlates with low and middle expression levels, yet not for high levels (Supplementary Fig S8 bottom). Finally, we provide this reviewer with the distribution of expression level for our study (cis genes) and Springer data set (Illustration 1). We do not think however that this plot is necessary for the manuscript itself and keep figure 4A.

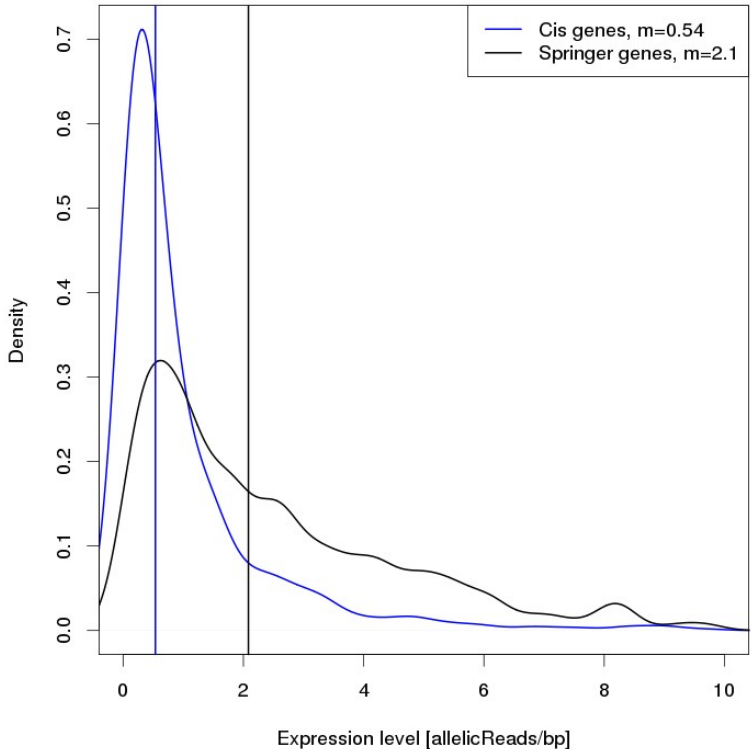

Illustration 1: Detailed Fig 4A. Density of expression level (allele specific reads per base pair) for the set of cis genes is compared against the genes used in the assay of Springer et al.(2010). The median expression level of the Springer set (black vertical line) is higher than the median of the cis genes (blue vertical line).

R2.4

In the Discussion (page 7) the authors state that "Hsp90 confers mutational robustness". This is a common mistake, but no experiment has yet been reported that tested whether Hsp90 buffers the effects of new mutations. Hsp90 impairment can reveal cryptic genetic variation, but this is not the same thing as lowering mutational robustness. Moreoever, in some cases Hsp90 impairment suppresses variation (leading to the inference that Hsp90 is a potentiator of variation rather than a capacitor). For an explanation of why cryptic genetic variation and mutational buffering are not necessarily related, see Hermisson & Wagner (2004). Also, for an argument against chaperones buffering mutations "by facilitating the correct folding of proteins" (as stated by the authors), see Bobula et al. (2006).

A: We thank this reviewer for pointing out this mistake and the references. We have removed the sentence “Hsp90 confers mutational robustness” as well as the statement about “chaperones conferring mutational robustness” (Discussion 4th paragraph).

R2.5

The Masel & Siegal (2009) review cited by the authors presents an argument about negative feedback and buffering environmental fluctuations vs. genetic variation that seems relevant to this manuscript. In particular, the review argues that negative feedback more generally buffers environmental fluctuations, but does not necessarily buffer genetic variation. But if the negative feedback operates through a highly cooperative mechanism then buffering of genetic variation would be more likely. Because the local trans variation seems not to be affecting transcription factor encoding genes, it might be difficult to test this idea about cooperativity but maybe the authors can think of something?

A: Indeed it is hard for us to assess the importance of cooperativity. One shall distinguish between robustness against mutations affecting the negative feedback loop itself from other regulatory mutations. Masel and Siegal (2009) consider robustness against mutations affecting the negative feedback loop itself (i.e. modeled by a change in the value of the repression constant r as defined in their Fig 1). Their mathematical model indicates that cooperativity confers robustness to such mutations. One reported instance of a mutation affecting a negative feedback loop is a coding mutation in the reference lab strain on the Amn1 protein (Ronald et al. 2005). In the case of such a mutated negative feedback loop, negative feedback is exerted only in the half of the spore population that inherited the genotype with functional feedback. Thus allelic differential expression is specific or at least stronger in the pool of spores than in the hybrid. Consistently, AMN1 showed only allelic differential expression in the spores in our data (Supplementary Fig S4). The new legend of supplementary figure S4 gives explanation about the interpretation of the allelic expression pattern in terms of mutated negative feedback loop.

Reviewer 3

Bader and colleagues have designed an elegant experimental systems to detect RNA expression buffering in hybrid strains. This systems compares allele expression in hybrid strains to those in a pool of spores. Using this approach the authors report their failure to detect signs of translation buffering described earlier and point at negative feedback as an important mechanism of allele buffering.

R3.1

Coming from an experimental background, I must confess that I had hard time getting my head around this paper. The experimental design is certainly elegant. However, the lack of follow up experiment makes it difficult for me to unequivocally endorse the authors' claims. My main problem is with "negative feedback" being pushed as a major mechanism of allele buffering. It is really not clear to me, for instance, how one can differentiate between negative feedback and another type of mechanism from the data alone in absence of functional follow up. This may be down to me not being an expert in the field, but many MSB readers won't be either. At the very least, the authors need to make it clearer why they believe their conclusions on the importance of negative feedback valid in absence of any functional confirmation.

A: Our claim is actually very general and include all kinds of auto-regulation that buffers regulatory variation. If not achieved by compensatory mutations, buffering of cis effects must be due to some auto-regulatory mechanism, which can be indirect. This auto-regulatory mechanism, has the overall effect of acting against deviations to a target equilibrium level, which defines negative feedback. Confirmation of auto-regulation is provided by the deletion screen (Springer et al. 2010 Molecular Systems Biology) on highly expressed, non-essential genes in line with our measures (Fig 4B). We understand that the reviewer would like to see functional follow-ups to get closer to molecular mechanisms, e.g. identifying at which level (transcription, RNA stability) or by what class of proteins this auto-regulation is actually achieved. The new version of the manuscript now reports further analyses to identify regulatory features enriched for buffered genes (2nd results section, 1st paragraph). Neither gene ontology categories nor features that have been associated with gene expression variability (TATA box) or dosage compensation in fly (gene length) showed significant association with the buffering coefficient (Supplementary Fig S5). We therefore keep a high-level claim of negative feedback including direct and indirect instances.

R3.2

Second, the authors reanalyze a ribosome profiling dataset and dismiss previous conclusions that adjustment of translation efficiency is playing an important role in allele buffering. Here Bader and colleague have the burden of proof. What are the evidence that their computational approaches are sensitive enough to detect the effects publishes previously? Ribosomal profiling data are quite different from RNA-seq data, regarding transcript coverage for instance, how does it impact on the sensitivity of their statistical analysis?

A: This is a fair concern. However, ribosomal profiling in hybrids turns out to be more reproducible between biological replicates than RNAseq levels of pools of spores (Supplementary Fig S1B compared to Artieri and Fraser Supplementary Fig S1). This higher reproducibility is certainly due to the added variation in independently (i) generating as well as (ii) growing two pools of spores. We are now making this point explicit in supplementary information (see legend Supplementary Fig S6). Nonetheless, the issue of sensitivity matters. Therefore, in our detailed reanalysis of Artieri's dataset (Supplement), we added estimates of a genome wide trend (by PCA in Supplementary Fig S6 or by distribution of buffering coefficients in Fig S12) which are not based on statistical tests on the ribosomal profiling data. Independently, part of our argumentation is also supported by a report that has been published since our first submission (Albert et al., 2014 PlosGen).

R3.3

From looking at figure 2C quite a few genes must have a C > 0, why are these not relevant?

A: Genes buffered by translational effects certainly occur but this is not the genome-wide trend (Fig 2B). We are now making explicit in the first paragraph of the discussion that our results are about the overall trend. Moreover, we make explicit now that translational buffering can occur in specific instances (discussion, 1st paragraph).

R3.4

I also have a couple of specific points that require clarification: Average allele specific reads per sample is around 10. I suppose that median number is lower. It is not clear to me how these relatively low numbers will affect conclusions in figure 3 where a relationship between transcripts expression levels and ADE is observed. What are the evidence that their approach is not biased by the number of diagnostic reads for each allele? This is also relevant for the ribosomal foot-printing data where numbers of allele specific reads are likely to be low.

A: We have clarified this point in the first results section second paragraph. The minimum average number of allele-specific reads per transcript is 10 (results “Dissecting cis- and local trans-regulatory effects”, second paragraph). Among them the number of allele-specifc reads is much larger (median 1044, now in the second paragraph of results).

R3.5

"Together, these findings indicate that buffering through local trans regulation had been underestimated so far.". It is not clear to me where previous estimates were published.

A: Previous local trans analysis was published by Ronald et al. (2005 Plos genetics), but indeed buffering through local trans regulation was not assessed. We changed this sentence to “local trans regulation might be frequent”.
